# Jolkinolide B Activates Mitophagy to Exhibit Antipancreatic Cancer Activity and Alleviate Cognitive Deficits in Alzheimer's Disease

**DOI:** 10.1016/j.mcpro.2025.101060

**Published:** 2025-08-25

**Authors:** Mingjie Gao, Weiyi Hu, Delan Meng, Pengju Yao, Siqi Yang, Yuanpeng Tong, Lei Wang, Ya Zhang, Qingsong Wang, Jianguo Ji, Wenyuan Zhu

**Affiliations:** State Key Laboratory of Gene Function and Modulation Research, School of Life Sciences, Peking University, Beijing, China

**Keywords:** mitophagy enhancer, pancreatic cancer, jolkinolide B, mitochondrial protein TOM40, Alzheimer's disease

## Abstract

Dysfunctional mitophagy leads to the pathological accumulation of damaged mitochondria, which is closely associated with the development of human diseases such as cancer and Alzheimer's disease. The identification of safer and more effective mitophagy regulators may provide a novel approach for treating mitochondrial diseases. Covalent-binding drugs have attracted substantial attention due to their high specificity, selectivity, and low resistance potential. In this study, we demonstrated that the natural epoxide compound jolkinolide B (JB) specifically induces mitophagy both *in vitro* and *in vivo*. Mass spectrometry analysis confirmed that JB directly binds to the outer mitochondrial membrane translocase protein TOM40, leading to autophagic cell death in pancreatic cancer. As a mitophagy enhancer, JB also ameliorates mitochondrial dysfunction and mitigates cognitive deficits in the 5×FAD mouse model of Alzheimer's disease. The findings indicate that JB selectively targets mitochondria to enhance mitophagy while exhibiting minimal toxicity in pancreatic cancer and Alzheimer's disease mouse models, highlighting its potential as a therapeutic agent for mitochondrial diseases.

## Highlights

• Quantitative proteomics shows that JB specifically enhances mitophagy.
• Mitochondrial protein TOM40 is identified as a covalent binding target of JB.
• JB blocks mitochondrial preprotein import and induces mitochondrial dysfunction.
• JB inhibits pancreatic cancer growth by inducing excessive autophagic cell death.
• JB restores mitophagy to improve cognitive defects in the 5×FAD Alzheimer's disease mouse model.

## Graphical Abstract

**Figure:**
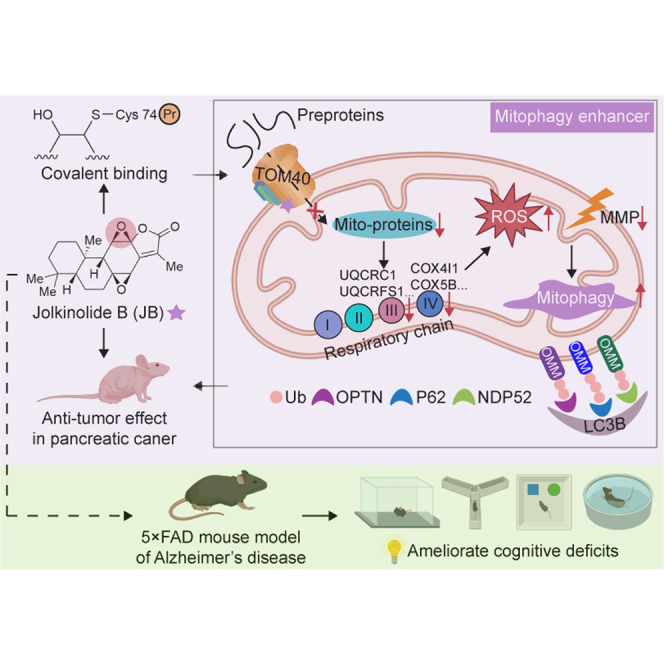

## In Brief

This study systematically elucidated the mechanism of natural compound jolkinolide B (JB), its binding targets and phenotype by using advanced proteomic techniques. We demonstrated that JB is a novel mitophagy enhancer and identified mitochondrial protein TOM40 as its previously unknown covalent target. JB exerts dual therapeutic efficacy in inhibiting pancreatic cancer and alleviating cognitive deficits in Alzheimer's disease, with a favorable safety profile. These findings provide theoretical support for designing new mitophagy-based drugs and advancing JB as a therapeutic candidate.

Mitochondria serve as the main energy-producing systems in most eukaryotic cells, generating ATP and various biosynthetic intermediates (1). However, mitochondria are highly susceptible to damage, which can lead to mitochondrial dysfunction—this process has been linked to many human diseases, including cancer, neurodegenerative disorders, and cardiovascular conditions (2). Notably, the selective removal of dysfunctional mitochondria (i.e., mitophagy) is an important pathway for maintaining mitochondrial balance and cellular homeostasis (3). Pancreatic cancer is among the most aggressive solid malignancies, characterized by low overall survival and poor response to existing therapies (4). Thus, novel and effective treatment strategies are urgently needed. There is evidence that pancreatic cancer has high basal autophagy levels, which are essential for maintaining cancer cell growth and survival (5). Paradoxically, although basal autophagy promotes cancer cell proliferation, excessive autophagy induces disproportionate removal of cellular components, ultimately leading to autophagic cell death (6). Indeed, some anticancer drugs exert cytotoxic effects by inducing excessive or persistent autophagy (7, 8). Alzheimer's disease (AD) is the most prevalent neurodegenerative disorder that currently lacks effective treatments (9). Emerging evidence suggests that disrupted mitophagy contributes to the pathological accumulation of dysfunctional mitochondria in the AD brain, a phenomenon that may drive memory impairment and dementia (10). Mitophagy restoration effectively inhibits disease progression in preclinical AD models (11, 12). Therefore, efforts to target mitophagy may represent promising treatment strategies for mitochondrial diseases, particularly through the development of activators or inducers with considerable therapeutic potential.

There is evidence that mammalian target of rapamycin (mTOR) plays important roles in cancer and neurodegenerative disorders, including AD (13). Currently, autophagy inducers in clinical trials primarily consist of mTOR inhibitors (e.g., sirolimus and its analogs), which have been approved by the United States Food and Drug Administration for the treatment of various malignancies (14, 15). In addition, sirolimus has been shown to abolish cognitive deficits and reduce amyloid-β levels in a mouse model of AD by inhibiting mTOR and activating mitophagy (16). However, its therapeutic applications in mitochondria-related diseases remain limited due to challenges such as poor specificity, serious side effects, and unclear molecular mechanisms (17, 18). Therefore, the development of selective autophagy activators with low toxicity and well-defined molecular mechanisms is particularly important for clinical applications. The successful development of targeted covalent inhibitors (TCIs) has demonstrated the feasibility of leveraging covalent bonding strategies for therapeutic purposes (19, 20, 21, 22, 23). Approximately 30% of marketed drugs function as covalent inhibitors (24), and these have been effectively utilized in the treatment of various diseases. Compared with reversible inhibitors, TCIs exhibit higher biochemical efficiency, stronger and more durable effects, and a lower potential for resistance (25). Notably, recent Food and Drug Administration-approved TCIs predominantly rely on acrylamide as the covalent warhead (26); alternative warheads have received less attention. Natural products play key roles in drug discovery due to their unique structural features and diversity (27). Among these, epoxides—a common class of bioactive compounds present in natural products—exhibit strong potential for covalent drug development through nucleophilic addition, which opens the epoxide ring to form covalent bonds. This approach provides a promising avenue for covalent drug development, introducing novel warheads and scaffolds distinct from acrylamide. Moreover, studies have demonstrated that certain natural bioactive drugs possess specialized mitochondrial-targeting capabilities (28), with minimal or no side effects, representing a new direction for the development of mitochondria-targeted treatment strategies.

Jolkinolide B (JB), a diterpenoid extracted from the roots of *Euphorbia*, contains two epoxy groups, making it a promising candidate for development as a novel small-molecule covalent drug. Recent studies have shown that JB exhibits significant antitumor activity against various cancers (29), including breast cancer (30), lung cancer (31), and colorectal cancer (32). However, the pharmacological targets that directly interact with JB remain unidentified, greatly hindering its further development and clinical application. Moreover, its potential antitumor effects in pancreatic cancer have not been explored, and its effects on neurodegenerative diseases remain unknown. In this study, we found that JB specifically enhances mitophagy to exert robust antitumor effects on pancreatic cancer *in vitro* and *in vivo*, with no obvious toxic side effects. In addition, we identified mitochondrial protein TOM40 as a direct target of JB, which disrupts mitochondrial function and induces autophagic cell death, contributing to its antitumor activity. Furthermore, JB treatment reverses cognitive defects in AD mice, improving learning and memory in the 5×FAD mouse model. These findings provide valuable theoretical insights for the design of novel treatment strategies for pancreatic cancer and AD, supporting the future development of JB as a therapeutic agent.

## Experimental Procedures

### Experimental Design and Statistical Rationale

To clarify the effect of JB in pancreatic cancer MIA PaCa-2 cells, we conducted tandem mass tag (TMT) labeling quantitative proteomic analysis on three samples treated with 0.1% dimethyl sulfoxide (DMSO) (control group) and three samples treated with 25 μM JB (experimental group). Bioinformatics analysis revealed that differentially expressed proteins (DEPs) were closely associated with the biological process of mitophagy. Subsequently, based on the characteristic that the active epoxy group in JB can form covalent bonds with cysteine residues in target proteins, we proposed a mass spectrometry (MS)-based strategy for detecting high-resolution modifications to directly identify binding targets. JB was utilized as a dynamic modification for target search and analysis, and we identified 26 proteins with JB modification. Through a combination analysis of forward and reverse strategy, mitochondrial protein TOM40 was identified as a highly credible potential target of JB, and a series of experiments confirmed that JB forms an irreversible covalent bond with TOM40-Cys74. Next, to investigate the functional impact of JB on its target protein TOM40, we conducted quantitative mitochondrial proteomics analysis using highly purified mitochondria. Mitochondria were isolated from three DMSO-treated samples and three JB-treated samples, followed by TMT-6 plex labeling for proteomic quantification. We found that JB disrupts TOM40 transport function, causing most mitochondrial proteins downregulation in the JB treatment group, especially those related to mitochondrial respiratory chain complexes Ⅲ and Ⅳ. Finally, we revealed that JB inhibits mitochondrial oxidative respiratory chain activity, leading to increased reactive oxygen species (ROS) generation and loss of mitochondrial membrane potential (MMP), thereby activating increased mitophagy. In the xenograft mouse model of pancreatic cancer, JB suppresses tumor growth by inducing excessive autophagic cell death. In the 5×FAD mouse model of AD, JB significantly alleviates cognitive and learning deficits in AD mice by repairing impaired mitophagy.

Proteomic MS data were analyzed using R packages in RStudio. Following the removal of nonidentified or missing abundance values, statistical analysis was performed using the edgeR package (version 4.2.2). Proteins with statistically significant changes (DEPs) were selected using the criteria of adjusted *p*-value (false discovery rate,FDR) < 0.05 and |Fold change| > 1.5 (i.e., upregulated >1.5 or downregulated <0.67). Then functional enrichment analysis and visualization were conducted on these DEPs.

Statistical analysis was performed using GraphPad Prism (version 8.0), and results are presented as mean ± standard error of the mean (s.e.m.). All experiments were independently repeated at least three times. Two-tailed unpaired Student's *t* test was used for comparisons between two groups; one-way analysis of variance (ANOVA) or two-way ANOVA was utilized for comparisons involving multiple groups. Statistical significance was denoted as follows: ∗*p* < 0.0332, ∗∗*p* < 0.0021, ∗∗∗*p* < 0.0002, and ∗∗∗∗*p* < 0.0001.

### Chemicals

Jolkinolide B (JB, purity ≥98%, CAS. No. 37905-08-1) and 17-hydroxy jolkinolide B (HJB, purity ≥98%, CAS. No. 116360-82-8) were purchased from Nanjing DASF Biotech Co, Ltd. HJB-Cy7 was synthesized by Xi'an RuixiBiotech Co, Ltd.

### Sample Preparation for Mass Spectrometry

MIA PaCa-2 cells were treated with 0.1% DMSO or 25 μM JB for 24 h. Protein lysates were obtained by adding 1% SDS. Protein samples were precipitated with acetone at −20 °C overnight, resuspended in 8 M urea, and sonicated using a Bioruptor. The samples were then incubated with DTT (Sigma-Aldrich, D5545) at room temperature for 30 min, with iodoacetamide (Sigma-Aldrich, I1149) in darkness for 30 min, and with DTT for an additional 10 min. Proteins were digested with Lys-C (1:100, w/w; Wako, 125-05061) for 3 h and then with trypsin (1:50, w/w; Promega, V5111) overnight at 37 °C. Digestion was terminated by adding 0.5% trifluoroacetic acid (TFA). Peptides were desalted using an Empore C18 column (CDS, 98-0604-0197-7), eluted with 0.1% TFA/70% acetonitrile (ACN), and vacuum-dried.

### TMT Labeling

Peptides were resuspended in 100 mM tetraethylammonium bromide, and peptide concentrations were measured using the Micro-BCA Protein Assay Kit (Thermo Fisher Scientific, 23235). Samples were labeled with 6-plex TMT (Thermo Fisher Scientific, 90068) and incubated at room temperature on a shaker for 1 h. Labeling was terminated by addition of 5% hydroxylamine to each tube. The labeled samples were mixed, vacuum-dried, resuspended in 0.1% TFA, and desalted again using a C18 column.

### HPLC and Liquid Chromatography Tandem Mass Spectrometry Analysis

Samples were dissolved in mobile phase A (ultrapure water containing 5% ACN and 0.1% TFA), then centrifuged at 12,000 rpm for 10 min; supernatants were collected for analysis using an UltiMate 3000 HPLC system. Sample analysis was performed with an Acclaim 120 C18 column (2.1 × 250 mm, 2.2 μm; Thermo Fisher Scientific, 074812). The flow rate was set at 0.3 ml/min, and the column temperature was maintained at 50 °C. Small molecules were eluted with 50%–100% mobile phase B (ultrapure water containing 90% ACN with 0.1% TFA) over 40 min, and peptides were eluted with 5%–90% mobile phase B over 120 min. Eluted peptides were fractionated into 12 fractions, vacuum-dried, and prepared for liquid chromatography tandem mass spectrometry. Samples were redissolved in 0.2% formic acid, loaded onto a C18 reverse-phase column, and separated using a Nano EASY-nLC 1200 liquid chromatography system (Thermo Fisher Scientific). Peptides were eluted at a flow rate of 300 nl/min with an ACN gradient of 5%–72%. The eluted peptides were analyzed using an Orbitrap Fusion Lumos Tribrid instrument (Thermo Fisher Scientific) in higher energy collision dissociation mode for ion fragmentation and data-dependent acquisition mode for data collection.

### Data Analysis and Bioinformatics Analysis

Raw MS data were processed using Proteome Discoverer (version 2.2, Thermo Fisher Scientific) with a customized UniProt human protein database (updated on 3/11/2021, 20,396 sequences). Trypsin (Full) was selected as the digestion enzyme, and the maximum number of missed cleavages allowed was set to 2. Precursor mass tolerance and fragment mass tolerance were set to 10 ppm and 0.02 Da, respectively. The FDR cutoff for both peptides and proteins was 0.01. For quantitative proteomics, TMT (N terminus and K) and carbamidomethyl (C) were specified as static modifications, whereas oxidation (M) and acetylation (protein N terminus) were defined as dynamic modifications. For target identification, oxidation (M), carbamidomethyl (C), acetylation (protein N terminus), and JB (C) were all regarded as dynamic modifications.

Quantitative MS data were further analyzed and visualized using RStudio (version 3.6.3). DEPs were identified using the edgeR package, with thresholds set at a fold change greater than 1.5 or less than 0.67, and an FDR below 0.05. Gene ontology (GO) analysis and gene set enrichment analysis were conducted using the clusterProfiler package in R. For spectral similarity analysis, the m/z and ionic intensity of all b and y ions identified in peptides were exported. The relative intensity of each ion was normalized to a maximum ionic intensity of 100. Spectral similarity was calculated using the SpectrumSimilarity function in the OrgMassSpecR package.

### Quantification of Autophagy

MIA PaCa-2 cells were seeded in a four-chamber 35-mm glass-bottom dish at a density of 10^5^ cells per well and treated with 25 μM JB or DMSO for 24 h. For the positive control, cells were treated with Earle's balanced salt solution (an autophagy inducer, Beyotime, C0213) for 4 h. Cells were then incubated with monodansylcadaverine (Beyotime, C3018) for 30 min at 37 °C and washed three times with assay buffer. Autophagosomes labeled with the green probe were observed using a fluorescence microscope (Dragonfly). Simultaneously, cells were collected, plated into a black 96-well plate at a density of 10^4^ cells per well and analyzed using a plate reader (BioTek) at 335 nm/512 nm excitation/emission.

### Transmission Electron Microscopy

MIA PaCa-2 cells were seeded in six-well plates containing clean glass sheets and treated with 25 μM JB or 0.1% DMSO for 6 or 24 h. Tumors from xenografted mice were harvested and cut into 1-mm^3^ cubes. Samples were fixed with 0.1 mol phosphate buffer (pH 7.4) containing 2.5% glutaraldehyde at room temperature for 1 h. Subsequently, 0.1 mol phosphate buffer containing 1% osmium tetroxide and 0.8% potassium ferrocyanide was added, and the samples were fixed for an additional 1 h in the dark. After three washes with water, samples were incubated with 1% uranyl acetate overnight at 4 °C. Samples were subjected to gradient dehydration with ethanol; they subsequently were embedded in resin, then sliced and placed onto copper grids. Autophagosomes and autolysosomes were observed using a 120-kV electron microscope (JEM-1400Flash) set to an accelerating voltage of 80 kV with 5000× or 20,000× magnification.

### Cell Culture

Human pancreatic cancer MIA PaCa-2 cells, human colon cancer HCT 116 and LoVo cells, human breast cancer MDA-MB-231 and MCF-7 cells, and human embryonic kidney 293T cells were purchased from American Type Culture Collection. Human pancreatic cancer BxPC-3 cells were generously provided by Professor Fuchou Tang (School of Life Sciences, Peking University). Human normal pancreatic epithelial HPDE6-C7 cells were kindly donated by Professor Xiaofeng Zheng (School of Life Sciences, Peking University). Human normal colon epithelial HIEC-6 and NCM460 cells were provided by Professor Peng Du (School of Life Sciences, Peking University). Most of the above cell lines were cultured in Dulbecco's modified Eagle's medium (HyClone, SH30243.01) supplemented with 10% fetal bovine serum (GeminiBio, 900-108); NCM460 cells were cultured in RPMI-1640 medium (Gibco, C11875500BT) with 10% fetal bovine serum (GeminiBio, 900-108). For protein expression, 293F cells were generously donated by Academician Fu Gao (Institute of Microbiology, Chinese Academy of Sciences) and cultured in SMM 293-TⅡ medium (Sino Biological, M293TⅡ) supplemented with 1% penicillin–streptomycin solution (HyClone, SV30010). All cell lines were maintained in a humidified incubator at 37 °C with 5% CO_2_.

### Cell Viability Assay

Cancer cell lines (MIA PaCa-2, BxPC-3, HCT 116, LoVo, MDA-MB-231, and MCF-7) and normal cell lines (HPDE6-C7, HIEC-6, and NCM460) were evenly seeded in 96-well plates at an appropriate density and cultured for 24 h to allow attachment. Cells were treated with the indicated concentrations of JB for 24, 48, or 72 h; 1% DMSO (Sigma-Aldrich, D2650) served as a control. After treatment, cells were incubated with cell counting kit-8 reagent (Beyotime, C0043) for 1 h; absorbance at 450 nm was then measured using a microplate reader (BioTek).

### Colony Formation Assay

MIA PaCa-2 cells and BxPC-3 cells were seeded in six-well plates and cultured for 48 h to allow attachment. Cells were then exposed to medium containing JB at concentrations of 0, 6.25, 12.5, 25, 50, or 100 μM for 5 days; medium containing fresh JB was replaced every 2 days. Cell status was monitored throughout the treatment period. Colonies were fixed with 4% fixative solution for 30 min, stained with crystal violet solution (Beyotime, C0121) for 15 min, and washed with PBS. Stained colonies were photographed using a microscope (Olympus IX71) and a phone camera, then counted using ImageJ software (version 2.14.0).

### Cellular Thermal Shift Assay

Cells treated with DMSO or 25 μM JB for 24 h were collected and washed twice with PBS. Cells were resuspended in PBS buffer containing 1% protease inhibitor cocktail (PI) and divided into 12 equal aliquots, each containing 10^6^ cells in 30 μl. Each aliquot was heated to the specified temperature (65, 70, 75, 77, 79, 81, 82, 83, 84, 85, 86, or 87 °C) for 3 min, then cooled to room temperature for 3 min. To each tube, 30 μl of cold PBS containing 0.2% Nonidet P-40 and 1% PI were added. The samples were immediately frozen and thawed three times to lyse the cells. Samples were centrifuged at 20,000*g* for 20 min at 4 °C to precipitate cell debris and aggregated proteins. Supernatants containing soluble proteins were carefully transferred to new tubes. Proteins were then separated and quantified via western blotting analysis.

### Protein Purification and Gel-Based Imaging

Codon-optimized DNA sequences of human *TOMM40* (UniProt: O96008) were cloned into the pcDNA3.1 vector with a C-terminal FLAG tag. The mutant *TOMM40*-C74S was generated by polymerase chain reaction amplification using the forward primer GCTGAAGATGGCGCAAGCGGTTGTCTGCCTAACCCCGGA and the reverse primer TGCGCCATCTTCAGCAGCGCCAG from the WT vector. Proteins were expressed in 293F cells cultured in SMM 293-TⅡ medium. Cells were resuspended in buffer containing 25 mM Tris (pH 7.8), 150 mM NaCl, 1.5% n-dodecyl β-D-maltoside, and 1% PI. After overnight incubation, lysates were centrifuged at 20,000 *g* for 45 min at 4 °C; the supernatant was filtered and incubated with anti-FLAG G1 affinity resin (Genscript, L00432-5) at 4 °C for 4 h. The resin was washed in a gravity column with 10 bed volumes of wash buffer (25 mM Tris, pH 7.8; 150 mM NaCl; 0.03% n-dodecyl β-D-maltoside). Proteins were eluted using wash buffer supplemented with 500 μg/ml FLAG peptide (GenScript) and concentrated to 400 μl using a 10 kDa cutoff centrifugal filter (Millipore, UFC901008).

For the irreversible covalent binding assay, recombinant WT TOM40 protein was preincubated with JB (0, 5, 50, or 500 μM) for 2 h at 4 °C, then incubated with 5 μM HJB-Cy7 at 4 °C for 2 h to allow competitive binding. Alternatively, proteins were incubated with or without JB (50 or 500 μM) at room temperature for 2 h, then incubated with 5-iodoacetamidofluorescein (5-IAF) (MedChemExpress, HY-D0807) at 37 °C for 30 min. For the binding specificity assay, WT or mutant C74S TOM40 protein was incubated with or without HJB-Cy7 for 2 h at 4 °C, or with or without 50 μM JB for 2 h at 4 °C, then labeled with 1 μM 5-IAF at 37 °C for 30 min. Samples were vortexed with loading buffer, subjected to 12% SDS-PAGE, and visualized using a ChemiDoc MP scanner (Bio-Rad). Silver staining (Thermo Fisher Scientific, 24600) was used as the loading control.

### Isolation of High-Purity Mitochondria

To obtain high-purity mitochondria for subsequent protein quantification analysis, we employed a combined method of differential centrifugation and Percoll density gradient centrifugation. MIA PaCa-2 cells treated with 0.1% DMSO or 25 μM JB were collected and resuspended in mitochondrial isolation buffer (10 mM Tris, pH 7.4; 1 mM EDTA-K_2_; 250 mM sucrose; and 1% PI). All steps were performed at 4 °C. Cells were disrupted using a homogenizer and centrifuged at 800*g* for 10 min to remove debris. The supernatant was collected and centrifuged at 21,000*g* for 10 min to isolate crude mitochondria, which were resuspended in 15% Percoll (Yeasen, 40501ES60). A Percoll gradient consisting of 15%, 23%, and 40% layers was carefully prepared using a flat-bottom needle; the sample was centrifuged at 30,700*g* for 20 min. The mitochondrial band located between the 23% and 40% Percoll layers was collected and transferred to a new ultrafiltration tube. The pellet was washed three times with mitochondrial isolation buffer to obtain high-purity mitochondria. The final mitochondrial sample was resuspended in 1% SDS with 1% PI and stored at −80 °C for further analysis. This approach aims to eliminate potential interference from cytoplasmic or other organelle proteins in MS or western blotting analyses, thereby ensuring the accuracy of protein quantification.

### Western Blotting

Proteins were denatured in 1 × SDS loading buffer by boiling for 5 min, separated on 10% or 12% SDS-PAGE, and transferred to polyvinylidene fluoride membranes (Bio-Rad, 40501ES60). Membranes were blocked with 5% skim milk dissolved in PBST (1× PBS containing 0.1% Tween 20) for 2 h at room temperature. Membranes were incubated with primary antibodies overnight at 4 °C, washed with PBST, and then incubated with secondary antibodies for 1 h at room temperature. After additional washes with PBST, immunoreactive bands were visualized using an ECL kit (Millipore, P90720). The antibodies used for this assay were listed in Supplemental Table S1.

### Detection of Complexes Ⅲ and Ⅳ Activity

To rapidly obtain mitochondrial fractions for the detection of complexes Ⅲ and Ⅳ activity, we strictly followed the standard protocol provided by the detection kits (Solarbio, BC3240; Acmec, AC10216). MIA PaCa-2 cells were seeded in 10-cm dishes at a density of 3 × 10^6^ cells/dish and treated with 25 μM JB or 0.1% DMSO for 24 h after attachment. Cells were then collected, resuspended in mitochondrial isolation buffer, and lysed using a glass homogenizer. After centrifugation at 600*g* for 10 min at 4 °C, the supernatant was transferred to a new tube and centrifuged again at 11,000*g* for 15 min at 4 °C to separate the cytoplasmic extracts (supernatant) and mitochondria (pellet). The mitochondrial pellet was resuspended in mitochondrial isolation buffer; the activity of complexes Ⅲ and Ⅳ was measured according to the respective kit protocols. This method focuses on preserving the conformation of enzyme proteins and avoiding activity loss due to excessive purification, making it suitable for the detection of complexes Ⅲ and Ⅳ activity.

### ROS Detection

MIA PaCa-2 cells treated with the indicated concentrations of JB, or shNC/shTOM40 cells, were seeded in six-well plates. Cells were incubated with 1 μmol/L 2′,7′-dichlorodihydrofluorescein diacetate (DCFH-DA) probe (Beyotime, S0033) at 37 °C for 20 min, washed with serum-free medium, and collected after digestion. Intracellular ROS levels were measured using flow cytometry (FACSVerse). Alternatively, cells were plated in a BeyoGold black 96-well plate at a density of 10^4^ cells/well (100 μl/well), and fluorescence was measured using a BioTek plate reader. The excitation wavelength was set to 488 nm, and the emission wavelength was set to 525 nm.

### MMP Detection

Cells were collected and resuspended in complete medium containing the JC-1 probe (Beyotime, C2003S). After incubation at 37 °C for 20 min, cells were washed twice with cold 1× JC-1 buffer. MMP was analyzed using FACSVerse flow cytometry, with FITC detecting JC-1 monomers and PE detecting JC-1 aggregates. For *in situ* detection, MIA PaCa-2 cells were seeded in a four-chamber 35-mm glass-bottom dish (10^5^ cells/well) and treated with 25 μM JB or DMSO for 24 h. JC-1 probe staining was performed directly in the dish, and samples were analyzed using a confocal microscope (Dragonfly). JC-1 monomers were detected using the 530/30 nm emission channel, and JC-1 aggregates were detected using the 590/35 nm emission channel. The objective oil lens was set to 63× magnification; the scale bar represents 50 μm.

### Animal Experiments

All animal experiments were approved by the Institutional Animal Care and Use Committee (IACUC) of the Center for Experimental Animal Research (Beijing, China) and the Peking University Laboratory Animal Center (Beijing, China; IACUC No. LSC-JiJG-9). Five-week-old female BALB/c nude mice were obtained from Beijing Vital River Laboratory Animal Technology Co, Ltd. MIA PaCa-2 cells (5 × 10^6^ cells in 0.1 ml PBS) were subcutaneously injected into the right flank of each mouse. When tumors reached a volume of 100 mm^3^, mice were randomly divided into four groups and given daily intraperitoneal injections of 20 mg/kg, 40 mg/kg, or 60 mg/kg JB (dissolved in PBS with 15% castor oil). Control mice received vehicle alone. Body weight and tumor volume were measured every 2 days during the 28-days experiment. Tumor volume (V) was calculated using the formula: V = 0.5 × length × width^2^. At the end of the experiment, mice were anesthetized via intraperitoneal injection of 2% tribromoethanol at a dose of 150 mg/kg, and blood was collected via retro-orbital bleeding, followed by centrifugation to obtain serum for liver and kidney function biochemical analysis. Following blood collection, mice were euthanized by cervical dislocation. Tumors were dissected and partially frozen for western blotting analysis and partially fixed for transmission electron microscopy imaging. The hearts, livers, spleens, lungs, and kidneys were dissected and collected for pathological sectioning, followed by H&E staining.

For the drug distribution assay, tumor-bearing mice were divided into a vehicle control group and a 60 mg/kg JB treatment group. After treatment for 4 h or continuous administration for 7 days, tumors and organs were harvested and washed with saline. Tissues were homogenized with ACN containing AMG 510 (MedChemExpress, CAS. No. 2296729-00-3) as an internal standard, then centrifuged at 12,000 rpm for 10 min at 4 °C; the supernatants were vacuum-dried. The content of JB was measured by HPLC, and the standard curves were generated using tissues from vehicle-treated mice.

### Transgenic Mice and Behavioral Studies

Subsequently, 5×FAD AD mice were obtained from the laboratory of Chen Zhang (School of Basic Medical Sciences, Capital Medical University). Male 5×FAD AD mice were mated with age-matched female C57/SJL mice, and male offspring were genotyped for experimental use. Male littermates with a negative genotype and the same genetic background served as WT controls. At 3 months of age, 5×FAD AD mice were randomized into two groups: one treated with JB (60 mg/kg/day, dissolved in PBS containing 15% castor oil; castor oil, MedChemExpress, HY-107799) and the other with vehicle control (PBS containing 15% castor oil), while WT mice received the same vehicle (PBS containing 15% castor oil) as blank controls. All treatments were administered via intraperitoneal injection for a 2-month period. At the study end point, behavioral assessments were first conducted, followed by anesthesia of mice via intraperitoneal injection of 150 mg/kg 2% tribromoethanol. Subsequently, cardiac perfusion was performed by sequentially infusing normal saline (to clear blood) and 4% paraformaldehyde (for tissue fixation) through the apex of the heart. Following perfusion, the whole brain was dissected, and hippocampal tissues were isolated for TEM analysis.

The open field test was used to evaluate autonomic behavior, anxiety-like behavior, and curiosity levels in mice (33). During the experiment, each mouse was placed in a designated location within a square arena, and activity was monitored for 10 min. Movement trajectories were tracked and data collection was performed using Activity Monitor software (version 7.8.0.0).

The Y-maze test was conducted to assess spatial recognition ability (34). The maze consisted of three arms (30 cm length × 8 cm width × 15 cm height). Each mouse was introduced to the center of the Y-maze and allowed to freely explore for 10 min. The Smart video tracking system (version 3.0.05) recorded arm entries and alternation patterns. Spontaneous alternation (%) was calculated as follows: number of actual alternations/(total arm entries − 2) × 100.

The novel object recognition test was performed as previously described (35). Mice were familiarized with the test environment (40 cm length × 40 cm width × 40 cm height) for 30 min on the day before the experiment. During the training phase, each mouse was allowed to explore two identical objects A for 10 min. After 1 h, one of the objects was replaced with a novel object B; after 24 h, a second novel object C was introduced. Exploration behavior was monitored using the Smart automatic tracking system (version 3.0.05). The recognition index was calculated as follows: time spent exploring novel object/total time spent exploring both objects.

The Morris water maze test was performed as previously described (36). Mice underwent training with four trials per day for four consecutive days. Each trial lasted 90 s or until the mouse located the platform. If the mouse failed to find the platform within the allotted time, the experimenter directed the mouse to the platform and allowed it to remain there for 10 s. After the training had been completed, the platform was removed on days 5 and 7 to assess spatial learning and memory. All parameters are recorded using the Morris water maze video tracking system.

## Results

### Quantitative Proteomics Reveals that JB Enhances Mitophagy in Pancreatic Cancer Cells

**Fig. 1:**
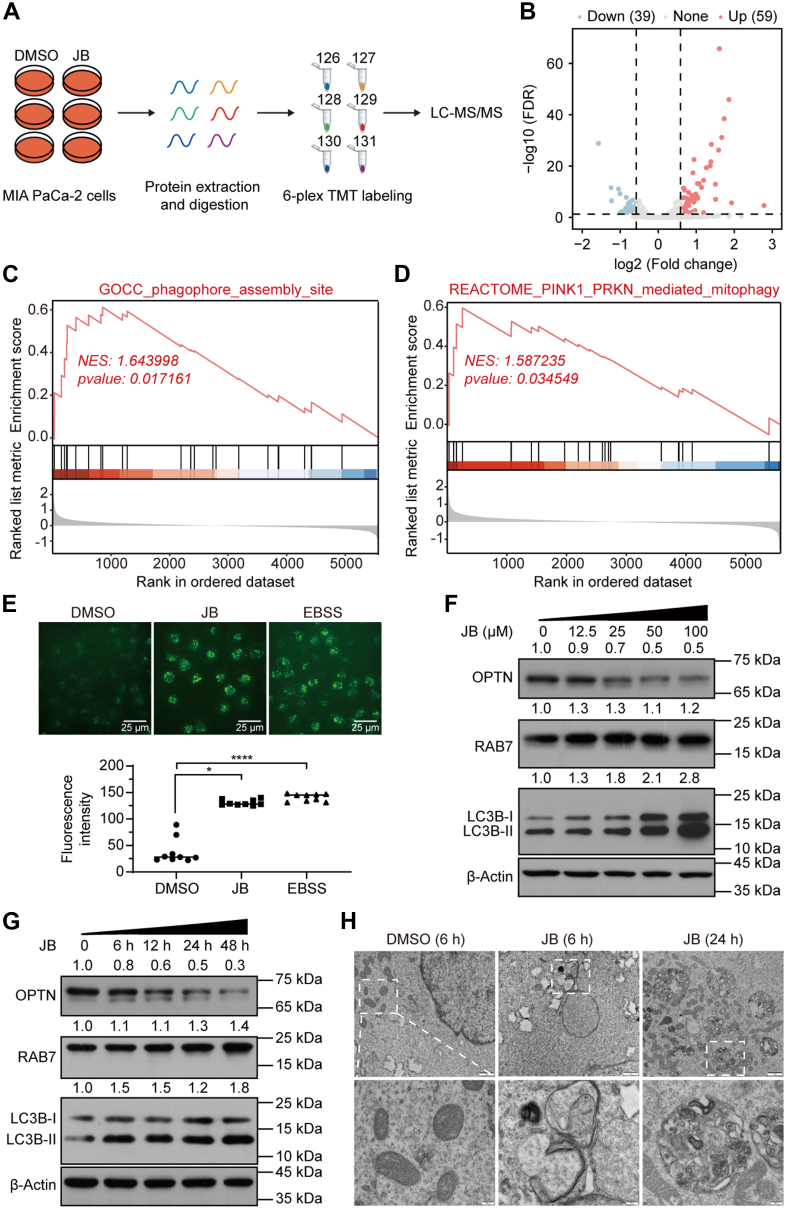
**Quantitative proteomics reveals that JB enhances mitophagy in pancreatic cancer cells.***A*, workflow of TMT-based quantitative proteomic profiling in MIA PaCa-2 cells treated with DMSO or 25 μM JB for 24 h. *B*, volcano plot showing the differential proteome of MIA PaCa-2 cells treated with JB. *Blue dots* (*down*) represent downregulated proteins, *red dots* (*up*) represent upregulated proteins, and *gray dots* (None) represent proteins with no change in expression. FDR: false discovery rate. *C*–*D*, gene set enrichment analysis (GSEA) of proteins in MIA PaCa-2 cells treated with JB relative to the DMSO group. NES: normalized enrichment score. *E*, MIA PaCa-2 cells were treated with DMSO, 25 μM JB for 24 h, or EBSS (positive control) for 4 h. Cells were then incubated with MDC and analyzed using a fluorescence microscope and a plate reader. Data are shown as mean ± standard error of the mean (s.e.m.) (n = 9 independent experiments). Statistical significance was determined by one-way analysis of variance (ANOVA). ∗*p* < 0.0332, ∗∗∗∗*p* < 0.0001. F–G, western blotting analysis of whole-cell lysates from MIA PaCa-2 cells treated with the indicated concentrations of JB for 24 h, or with 25 μM JB for 0, 6, 12, 24, or 48 h. *H*, transmission electron microscopy images of mitophagy in MIA PaCa-2 cells. The scale bars represent 1 μm (*upper* panel) and 200 nm (*lower panel*). EBSS, Earle's balanced salt solution; DMSO, dimethyl sulfoxide; JB, jolkinolide B; MDC, monodansylcadaverine; TMT, tandem mass tag.

Target prediction based on phenotypic changes and pathway enrichment analysis is an established forward strategy for identifying the molecular targets of natural products (37). To elucidate the effect of JB in pancreatic cancer, we analyzed proteomic changes in MIA PaCa-2 cells after 24 h of JB treatment using TMT labeling quantitative proteomics (Fig. 1*A*, Supplemental Table S2 and S6). In total, 98 DEPs were identified, including 59 upregulated proteins and 39 downregulated proteins (Fig. 1*B*). GO analysis revealed that these DEPs were significantly enriched in pathways related to cellular catabolic regulation, autophagy, and oxidative stress (Supplemental Fig. S1*A*). In addition, GSEA indicated that JB treatment was strongly linked to phagophore assembly and PINK1-PRKN-mediated mitophagy (Fig. 1, *C* and *D*). These findings suggest that JB can regulate autophagy levels in pancreatic cancer MIA PaCa-2 cells, a process closely associated with mitochondrial function.

To further explore the effect of JB on autophagy, we used monodansylcadaverine staining to detect autophagosome formation. JB treatment significantly increased autophagy in MIA PaCa-2 cells (Fig. 1*E*), producing effects similar to those of Earle's balanced salt solution starvation, which served as a positive control for autophagy induction. Next, we conducted western blotting analysis to assess protein markers associated with autophagy. JB treatment increased levels of the autophagy markers LC3B-Ⅱ and RAB7 in a dose- and time-dependent manner in both MIA PaCa-2 cells (Fig. 1, *F* and *G*) and BxPC-3 cells (Supplemental Fig. S1, *B* and *C*). Notably, JB treatment did not affect autophagy levels in normal pancreatic epithelial HPDE6-C7 cells (Supplemental Fig. S1, *D* and *E*). The LC3-interacting region plays a critical role in mechanisms of selective autophagy, including mitophagy. The reduced levels of autophagy adaptor proteins OPTN observed after JB treatment indicated completion of autophagic flux or autophagic degradation (Fig. 1, *F* and *G*, Supplemental Fig. S1, *B* and *C*). TEM imaging confirmed these findings, revealing that JB-treated cells contained cytoplasmic components encapsulated within double-membrane autophagosomes and exhibited an increased number of autolysosomes formed through lysosome fusion (Fig. 1*H*). These results suggest that JB enhances mitophagy in pancreatic cancer cells, consistent with functional annotations of the differential proteome.

### JB Suppresses the Proliferation of Pancreatic Cancer Cells with Minimal Toxicity to Normal Cells

**Fig. 2:**
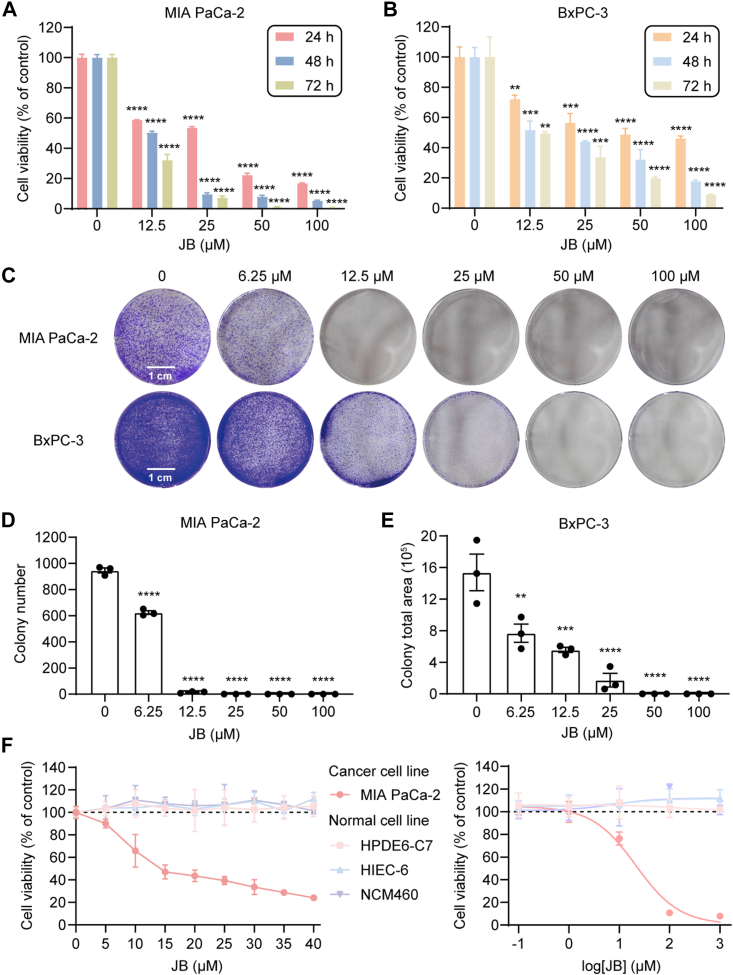
**JB suppresses cell proliferation of pancreatic cancer cells without affecting normal cells.***A*–*B*, viabilities of MIA PaCa-2 and BxPC-3 cells treated with JB (0, 12.5, 25, 50, and 100 μM) for 24, 48, or 72 h, as determined via CCK-8 assay. *C*–*E*, clonal survival of MIA PaCa-2 and BxPC-3 cells treated with JB (0, 6.25, 12.5, 25, 50, and 100 μM) for 5 days, as measured by colony formation assay. Colony number and total area were quantified using ImageJ software. The scale bar represents 1 cm. *F*, viabilities of pancreatic cancer cells (MIA PaCa-2), human normal pancreatic epithelial cells (HPDE6-C7), and normal colon epithelial cells (HIEC-6 and NCM460) treated with the indicated concentrations of JB for 24 h, as determined by CCK-8 assay. All data are presented as mean ± s.e.m. of three independent experiments. Statistical significance was determined using one-way ANOVA. ∗∗*p* < 0.0021, ∗∗∗*p* < 0.0002, ∗∗∗∗*p* < 0.0001. CCK-8, cell counting kit-8; JB, jolkinolide B.

To investigate the antitumor effects of JB on pancreatic cancer, cell viability was assessed using the cell counting kit-8 assay. JB substantially reduced the viability of multiple cancer cell lines (Supplemental Fig. S2, *A* and *B*, Supplemental Table S3), consistent with its previously reported broad-spectrum antitumor effects (38). To our knowledge, this study is the first to demonstrate that JB inhibits the proliferation of human pancreatic cancer cell lines MIA PaCa-2 and BxPC-3 (Supplemental Fig. S2, *C* and *D*) in a dose- and time-dependent manner. The 50% effective concentrations (EC_50_) at 24 h were approximately 25 μM for MIA PaCa-2 and BxPC-3 cells (Fig. 2, *A* and *B*). Subsequently, colony formation assays showed that JB reduced both the number and size of colonies formed by MIA PaCa-2 and BxPC-3 cells in a dose-dependent manner (Fig. 2, *C*–*E* and Supplemental Fig. S2*E*), indicating its ability to inhibit long-term proliferation. Notably, JB exhibited minimal toxicity to the normal human pancreatic epithelial cell line HPDE6-C7 (Fig. 2*F*), even at high concentrations, and demonstrated similarly low toxicity in other normal cell lines.

### Mass Spectrometry Identifies TOM40 as a Potential Target of JB

**Fig. 3:**
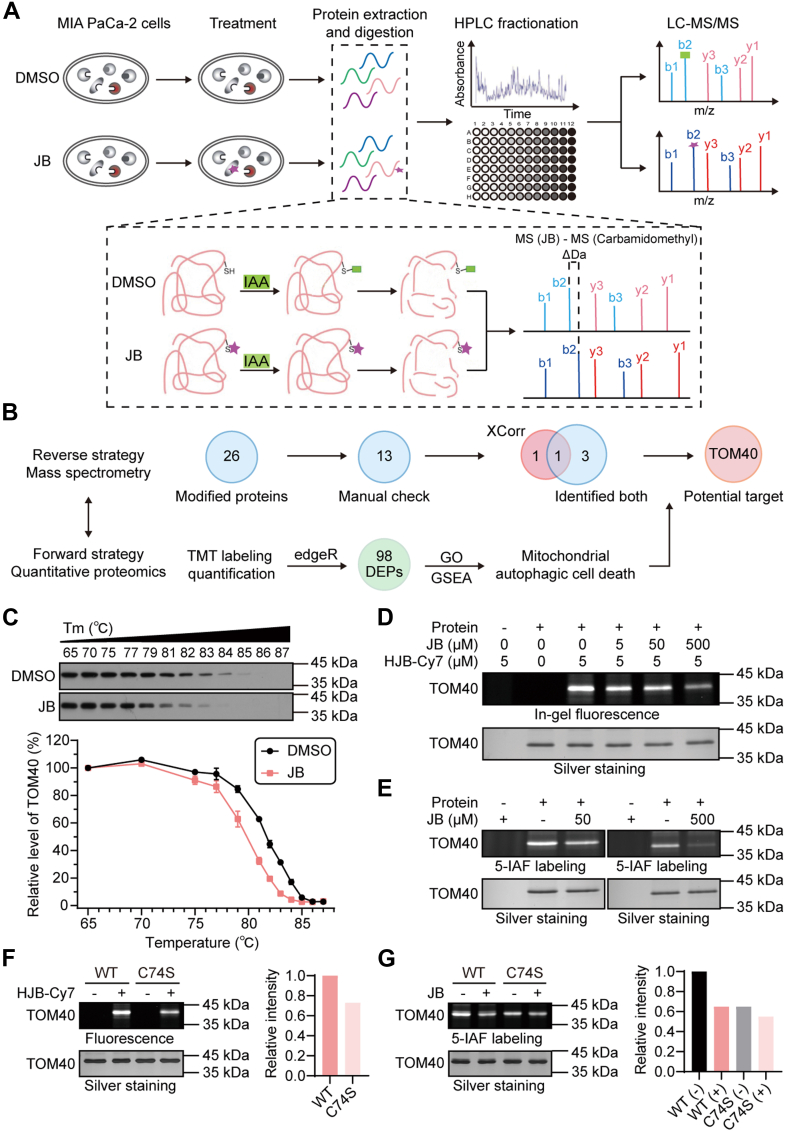
**Mass spectrometry directly identifies TOM40 as a covalent binding target of JB.***A*, workflow for the direct identification of JB targets using mass spectrometry. MIA PaCa-2 cells were treated with 25 μM JB or DMSO for 24 h. Iodoacetamide (IAA) served as an alkylating reagent for cysteine; the *purple star* represents the JB molecule. *B*, schematic illustrating the combined analysis of forward and reverse strategies used to screen potential JB targets. XCorr represents the reliability score of the spectrum; Identified both refers to peptides with carbamidomethyl modification in the DMSO group and JB modification in the JB treatment group. TMT: tandem mass tag; DEPs: differentially expressed proteins; GO: gene ontology. *C*, cellular thermal shift assay (CETSA) was conducted to measure the binding affinity of JB to TOM40 in MIA PaCa-2 cells. Data represent mean ± s.e.m. from at least three independent experiments. *D*, recombinant TOM40 protein was preincubated with JB (0, 5, 50, or 500 μM) for 2 h, then incubated with 5 μM HJB-Cy7 for competitive binding. *E*, recombinant TOM40 protein was incubated with or without JB (50 or 500 μM) for 2 h, and 5-IAF was used to label cysteine residues on TOM40. *F*–*G* WT TOM40 protein and mutant C74S protein were incubated with HJB-Cy7 or JB. Labeled proteins were scanned using the ChemiDoc MP imaging system, and silver staining was performed as a loading control. 5-IAF, 5-iodoacetamidofluorescein; DEP, differentially expressed protein; DMSO, dimethyl sulfoxide; HJB, 17-hydroxy-jolkinolide B; JB, jolkinolide B.

Several approaches have been developed to identify potential targets of small molecules, based on affinity or structural stability between drugs and targets (39). However, these methods often face limitations when applied to natural products with complex structures and multiple targets. This challenge emphasizes the need to develop high-throughput and efficient methods for direct target identification. To address this issue, we proposed a novel MS-based strategy for identifying small-molecule targets (Fig. 3*A*). By leveraging the reactive epoxy group in JB, which can form covalent bonds with cysteine residues in target proteins, we used JB (330.183 Da) as a dynamic modification for target search and analysis. This approach identified 26 proteins with cysteine residues containing the JB modification. To prioritize the most promising targets for further investigation, we applied multiple screening steps (Fig. 3*B*). Our manual curation of spectra quality identified 13 well-matched modified proteins (Table 1). Among these, TOM40 and DTX1 displayed high XCorr scores. In addition, TOM40, QCR6, ZNF24, and MALT1 were detected in both DMSO and JB-treated samples (Table 2).

**Table 1:** Proteins and peptides with JB modifications exhibiting high confidence

Protein	Annotated sequence	Modifications
TOM40	[R].TPGAATASASGAAEDGAc GcLPNPGTFEEcHR.[K]	C18 (JB); C20 (※); C30 (※)
QCR6	[R].LELcDER.[V]	C4 (JB)
DTX1	[K].DGSLQcPTCK.[TA]	C6 (JB)
ZNF24	[K].YGETcFPK.[G]	C5 (JB)
NID2	[R].cHPAATcYNTPGSFSCR.[C]	C1 (JB); C7 (※)
LTBP4	[K].APVLcPLIcHNGGVcVKPDR.[C]	C5 (JB); C9 (JB); C15 (※)
MALT1	[R].LScLDLEQcSLK.[V]	C3 (JB); C9 (※)
PKHA8	[K].STcNTFLK.[T]	C3 (JB)
EXOSX	[K].LYcNVDSNK.[Q]	C3 (JB)
VP13A	[K].NcILDDK.[R]	C2 (JB)
YD021	[K].TLKDcR.[I]	C5 (JB)
CA094	[K].KPTcPAEK.[N]	C4 (JB)
GASR	[R].CcPRPPR.[A]	C2 (JB)

※ Represents the carbamidomethyl modification.

**Table 2:** Key information used for screening potential targets

Protein	Charge	XCorr	Identified both	Location
TOM40	3	**5.42**	**Yes**	Outer mitochondrial membrane
QCR6	2	1.48	**Yes**	Inner mitochondrial membrane
DTX1	2	**2.37**	No	Cytoplasm, nucleus
ZNF24	2	2.12	**Yes**	Nucleus
NID2	4	1.93	No	Secreted protein
LTBP4	3	1.90	No	Secreted protein
MALT1	3	1.59	**Yes**	Cytoplasm, nucleus
PKHA8	2	1.35	No	Golgi apparatus
EXOSX	2	1.09	No	Cytoplasm, nucleus
VP13A	2	1.02	No	Membrane protein
YD021	2	0.94	No	Unknown
CA094	2	0.78	No	Unknown
GASR	2	0.75	No	Cell membrane

The reliability criteria for protein identification by mass spectrometry are as follows: charge = 2, XCorr >2.2; charge = 3, XCorr >3.75. XCorr: reliability score of the spectrum. Entries in bold represent high-confidence peptides that were alkylated (carbamidomethylated) in the DMSO control group and modified by JB in the JB treatment group.

To enhance reliability and reduce the workload involved in target validation, we proposed a combined approach comprising forward and reverse strategies. Differential phenotypic analysis indicated that the target of JB is closely associated with mitochondria, providing valuable guidance for target identification. Through comprehensive analysis, we identified the mitochondrial protein TOM40 as a highly credible potential target of JB. Further examination of the secondary spectrum of the JB-modified peptide from TOM40 revealed a nearly complete set of b ions and y ions (Supplemental Fig. S3*A*, Supplemental Table S4). The observed mass difference of the parent ion (273.164 Da) closely matched the theoretical mass difference (273.162 Da), confirming the reliability of this MS-based target identification approach. RNA expression database analysis indicated that TOM40 is highly expressed in pancreatic tumor tissues (Supplemental Fig. S3*B*) consistent with the characteristic active mitochondrial metabolism in cancer cells (40). Moreover, we demonstrated that TOM40 protein expression is higher in cancer cells than in normal cells (Supplemental Fig. S3*C*), supporting its research value as a potential drug target.

### Cys74 Is the Key Residue for JB-TOM40 Covalent Binding

To confirm the stability of the covalent bond between JB and TOM40, we performed an immunoprecipitation-mass spectrometry assay to enrich TOM40 protein (Supplemental Fig. S4*A*). After immunoprecipitation enrichment, we observed a substantial increase in the number of peptides and peptide spectrum matches (PSMs); we identified 253 PSMs corresponding to 17 peptides (Supplemental Fig. S4*B*). JB modification at cysteine 74 (Cys74) on TOM40 was detected again, consistent with the results of whole-cell lysate analyses. Furthermore, a greater number of the same modification spectra were identified in the enrichment group; six PSMs were detected in the two experimental groups. These spectra displayed improved quality, with an XCorr score of 8.25 (Supplemental Fig. S4*B*) and a spectral similarity of 0.9832381 (Supplemental Fig. S4*C*). Subsequent cellular thermal shift assay demonstrated that JB reduced the thermal stability of TOM40 in MIA PaCa-2 cells (Fig. 3*C*) and BxPC-3 cells (Supplemental Fig. S4*D*), confirming a direct interaction between JB and TOM40.

To explore the specificity of the interaction between JB and TOM40-Cys74, we performed an *in vitro* incubation assay with recombinant protein. Because JB lacks intrinsic reactive groups, we utilized a structurally similar compound, HJB, to synthesize the fluorescent probe HJB-Cy7 (Supplemental Fig. S4*E*). Recombinant TOM40 protein was incubated with increasing concentrations of JB, followed by HJB-Cy7. Comigration of HJB-Cy7 and TOM40 on a denaturing gel indicated covalent linkage between them. As shown in Figure 3*D*, fluorescence intensity decreased as JB concentrations increased, confirming irreversible covalent binding between JB and TOM40. In addition, compared with the DMSO control, JB reduced 5-IAF labeling on TOM40 (Fig. 3*E*), demonstrating that JB modifies TOM40 through the cysteine residue. To validate the interaction between JB and TOM40-Cys74, the mutant protein (C74S) was expressed and purified *in vitro*. Compared with the WT protein, fluorescence intensity of the C74S mutant with HJB-Cy7 was substantially reduced (Fig. 3*F*), and JB did not affect 5-IAF labeling of the C74S mutant (Fig. 3*G*). These results confirmed that Cys74 is the primary binding site for JB on TOM40. Moreover, JB did not alter TOM40 expression levels but caused a slight mass shift due to covalent binding (Supplemental Fig. S4*F*). This mass shift was also observed in tumor samples from JB-treated mice (Supplemental Fig. S4*G*).

### JB Disrupts TOM40 Transport Function, Causing Mitochondrial Preprotein Import Disorder

**Fig. 4:**
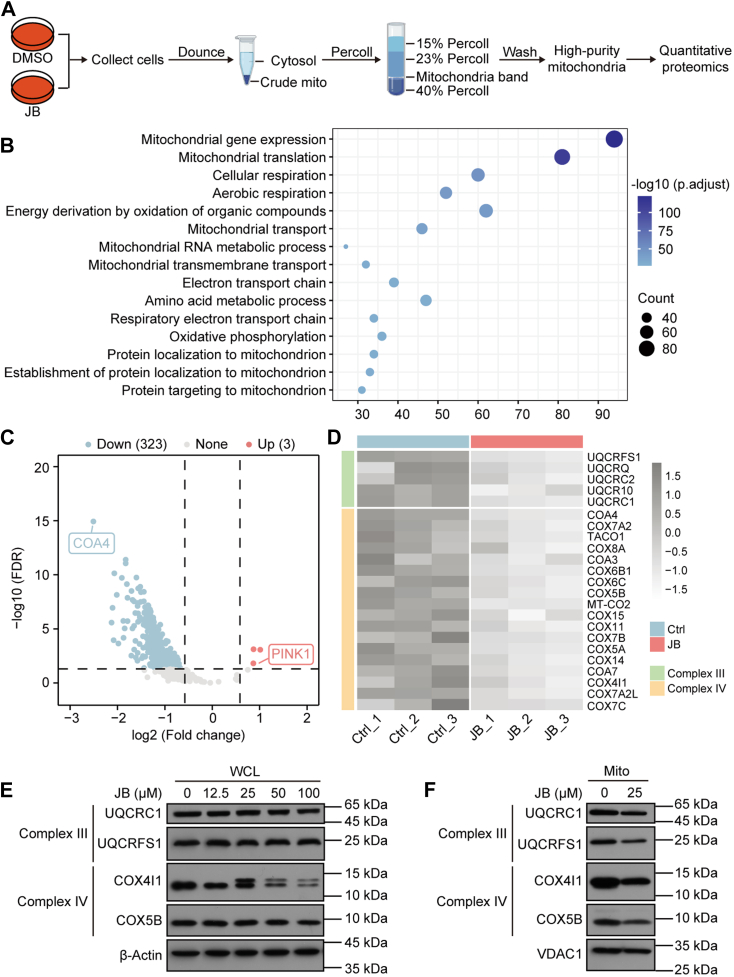
**JB blocks the import of many precursor proteins into mitochondria.***A*, workflow illustrating the isolation of high-purity mitochondria for quantitative proteomics analysis. *B*, biological process annotation enrichment analysis of DEPs in mitochondria. *C*, volcano plot visualizing mitochondrial proteins. Upregulated proteins are shown in *red* (Up; Fold change >1.5, FDR <0.05), and downregulated proteins are shown in *blue* (Down; Fold change <0.67, FDR <0.05). *D*, heatmap showing expression levels of respiratory chain complex Ⅲ and complex Ⅳ subunit proteins in mitochondria from the DMSO-treated group (Ctrl) and JB-treated group (JB). *E*–*F*, western blotting analysis of mitochondrial respiratory chain component subunit expression. Analysis of whole-cell lysates (WCLs) using β-Actin as the loading control (*E*), and mitochondrial fractions (Mito) using VDAC1 as the loading control (*F*). DEP, differentially expressed protein; DMSO, dimethyl sulfoxide; FDR, false discovery rate; JB, jolkinolide B.

We next investigated how JB affects the function of its target TOM40. TOM40 is the primary channel subunit of the outer mitochondrial membrane translocase (TOM complex) (41), which is essential for importing preproteins into mitochondria. Approximately 99% of mitochondrial precursor proteins are encoded by nuclear genes, synthesized in the cytoplasm, and transported into mitochondria via the TOM complex (42). To determine whether JB affects the ability of the TOM complex to transport precursor proteins into mitochondria, we isolated high-purity mitochondria for quantitative analysis (Fig. 4*A*, Supplemental Fig. S5*A*, Supplemental Table S5 and S6). In total, 470 mitochondrial proteins were identified, of which 326 were DEPs. GO annotation revealed that these DEPs were primarily enriched in processes related to mitochondrial gene expression, mitochondrial transport, and respiratory electron transport chain (Fig. 4*B*). Most mitochondrial proteins were significantly downregulated (Fig. 4*C*), particularly those associated with mitochondrial respiratory chain complexes Ⅲ and Ⅳ (Fig. 4*D*). Western blotting analysis confirmed that the expression levels of these respiratory chain subunit proteins were significantly reduced in mitochondria but remained unchanged in whole-cell lysates (Fig. 4, *E* and *F*), suggesting that JB inhibits the import of precursor proteins into mitochondria. In addition, based on the TOM complex assembly model proposed by Wang *et al*. (43), we observed that JB may impair the dissociation of TOM40 from the SAM complex (Supplemental Fig. S5, *B* and *C*), thereby disrupting TOM complex assembly and its transport function.

### JB Induces Mitochondrial Dysfunction by Inhibiting Respiratory Chain Activity

**Fig. 5:**
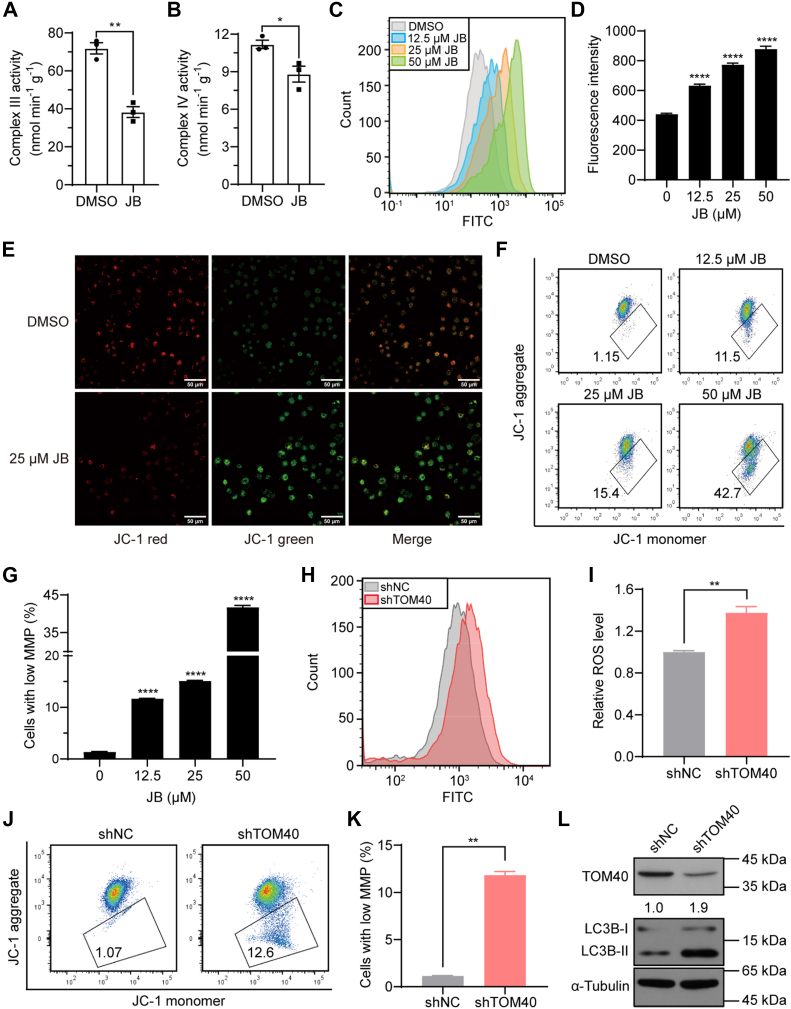
**JB inhibits respiratory chain activity to induce mitochondrial dysfunction associated with TOM40 deficiency.***A*–*B*, mitochondria were isolated, and the activity of respiratory chain complexes Ⅲ and Ⅳ was measured using a spectrophotometer to record absorbance at 550 nm. *C*–*D*, MIA PaCa-2 cells were treated with JB (0, 12.5, 25, or 50 μM) for 24 h. ROS levels were labeled with the DCFH-DA probe and detected using flow cytometry (*C*) and a microplate reader (*D*). *E*-*G*, MIA PaCa-2 cells treated with the indicated concentrations of JB for 24 h were incubated with the JC-1 probe to label mitochondrial membrane potential (MMP). MMP was detected using fluorescence microscopy (*E*; scale bar represents 50 μm) and flow cytometry (*F*, *G*). *H*–*I*, ROS levels in shNC and shTOM40 cells were measured using the DCFH-DA probe and detected by flow cytometry. *J* and *K*, MMP in shNC and shTOM40 cells was labeled with the JC-1 probe and detected by flow cytometry. *L*, western blotting analysis was performed to determine autophagy levels in shNC and shTOM40 cells. Experiments were repeated at least three times. Data are presented as mean ± s.e.m. Statistical significance was determined using two-tailed unpaired Student’s *t* test (*A*, *B*, *I*, and *K*) or one-way ANOVA (*D*, *G*). ∗*p* < 0.0332, ∗∗*p* < 0.0021, ∗∗∗∗*p* < 0.0001. JB, jolkinolide B; ROS, reactive oxygen species.

Subsequently, we evaluated the activity of the mitochondrial respiratory chain and found that JB significantly inhibited the activity of complexes Ⅲ and Ⅳ (Fig. 5, *A* and *B*). Impairment of the mitochondrial electron transport chain is believed to increase the production of ROS; complex Ⅲ serves as a primary source of ROS (44). Using the DCFH-DA probe to assess redox status, we observed a dramatic, concentration-dependent increase in ROS production after JB treatment (Fig. 5, *C* and *D*). MMP homeostasis is essential for normal mitochondrial function. To assess MMP in JB-treated cells, we utilized the JC-1 probe. Representative images showed that exposure of MIA PaCa-2 cells to JB caused a shift in JC-1 fluorescence to green, indicating monomer formation and loss of MMP (Fig. 5*E*). Flow cytometry analysis further demonstrated a significant increase in the proportion of cells with mitochondrial depolarization after JB treatment (Fig. 5, *F* and *G*). Excessive ROS accumulation and MMP loss act as key signals in mitophagy activation (45). These results suggest that JB disrupts mitochondrial respiratory chain function by inhibiting the import of precursor proteins through TOM40. This disruption leads to increased ROS production and MMP loss, ultimately triggering mitochondrial autophagic death in pancreatic cancer cells.

To explore whether JB-mediated antiproliferation depends on TOM40, we generated TOM40-knockdown MIA PaCa-2 cells (shTOM40), using an empty vector as a negative control (shNC) (Supplemental Fig. S6*A*). The results showed that shTOM40 cells exhibited significantly reduced proliferation compared with shNC cells (Supplemental Fig. S6, *B*–*D*). Next, we investigated mitochondria-related functions in shTOM40 and shNC cells. TOM40 knockdown led to increased ROS accumulation (Fig. 5, *H* and *I*) and MMP loss (Fig. 5, *J* and *K*), along with enhanced autophagy (Fig. 5*L*). These effects were consistent with those observed in JB-treated MIA PaCa-2 cells. Our findings demonstrate that JB induces autophagic cell death in pancreatic cancer cells by impairing TOM40-related functions.

### JB Inhibits Tumor Growth in Pancreatic Cancer by Inducing Autophagic Cell Death

**Fig. 6:**
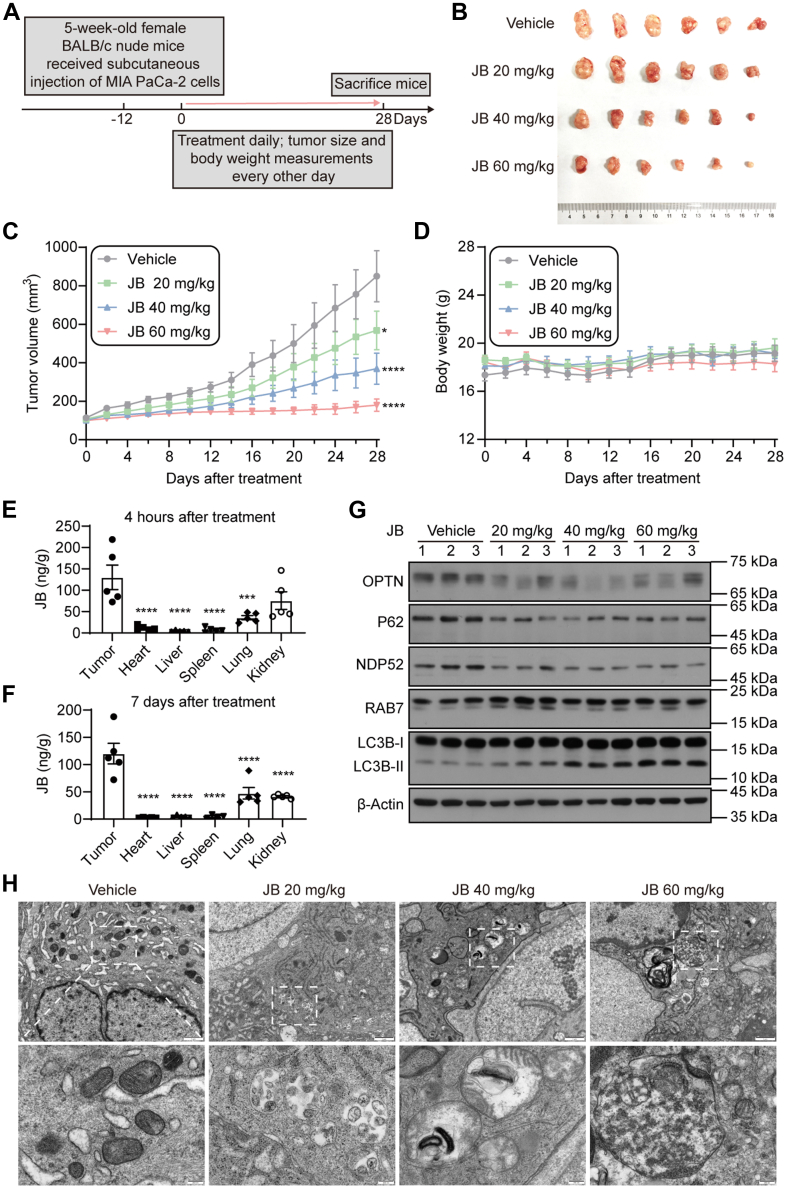
**JB inhibits tumor growth in a pancreatic cancer xenograft mouse model with no obvious toxic effects.***A*, schematic workflow for evaluating the *in vivo* antitumor effects of JB. MIA PaCa-2 cells were implanted into the right subcutaneous flanks of BALB/c nude mice. Tumor-bearing mice were intraperitoneally injected with JB (20, 40, or 60 mg/kg) or vehicle (DMSO) daily for 28 days. *B*, photograph of dissected tumors at the end of the experiment. *C*, tumor volume curves measured at different time points during the treatment period (n = 6 per group; mean ± s.e.m.; two-way ANOVA). ∗*p* < 0.0332, ∗∗∗∗*p* < 0.0001. *D*, graph showing body weights of tumor-bearing mice throughout the treatment period (n = 6). *E*–*F*, JB content in tumors and normal organs of tumor-bearing mice was measured after 4 h or continuous administration for 7 days. The JB content was calculated as JB (ng) per tissue weight (g) (n = 5 per group; mean ± s.e.m.; one-way ANOVA). ∗∗∗*p* < 0.0002, ∗∗∗∗*p* < 0.0001. *G*, western blotting analysis of tumor tissues harvested from xenografted mice. *H*, tumor tissues from xenografted mice were imaged using transmission electron microscopy. The scale bars represent 1 μm (*upper panel*) and 200 nm (*lower panel*). DMSO, dimethyl sulfoxide; JB, jolkinolide B.

To evaluate the antitumor potential of JB *in vivo*, we established a xenograft mouse model by subcutaneously injecting pancreatic cancer MIA PaCa-2 cells into the right flanks of experimental mice. When tumors reached a volume of 100 mm^3^, mice were randomly divided into four groups and intraperitoneally injected with either vehicle (DMSO) or JB (20 mg/kg, 40 mg/kg, or 60 mg/kg) daily for 28 days (Fig. 6*A*). As shown in Figure 6*B*, JB significantly inhibited tumor growth at the 60 mg/kg dose compared with the control group. Consistent with this finding, JB treatment reduced the tumor growth rate in a dose-dependent manner (Fig. 6*C*). Importantly, JB was well-tolerated in mice; no significant weight loss was observed relative to the control group (Fig. 6*D*). Similarly, H&E staining of organs from the control and JB-treated groups revealed no significant histological abnormalities (Supplemental Fig. S7*A*). In addition, serum biochemical analyses showed no differences among the groups (Supplemental Fig. S7, *B*–*G*), indicating that JB did not cause detectable liver or kidney damage. Efficient drug distribution to the target site is essential for enhancing efficacy and minimizing side effects (46). Accordingly, we used HPLC to measure JB content in various organs. The results indicated that JB accumulation was greater in tumors than in normal organs (e.g., heart, liver, spleen, lung, and kidney) (Fig. 6, *E* and *F*). In addition, JB-induced increased mitophagy was confirmed in tumor tissues collected from JB-treated mice (Fig. 6, *G* and *H*), demonstrating that the mechanism identified *in vitro* also occurs in a xenograft mouse model of pancreatic cancer. Collectively, these findings demonstrate that JB effectively inhibits pancreatic cancer *in vitro* and *in vivo* while exhibiting low toxicity.

### JB Restores Mitophagy to Ameliorate Cognitive Defects in the 5×FAD AD Mouse Model

**Fig. 7:**
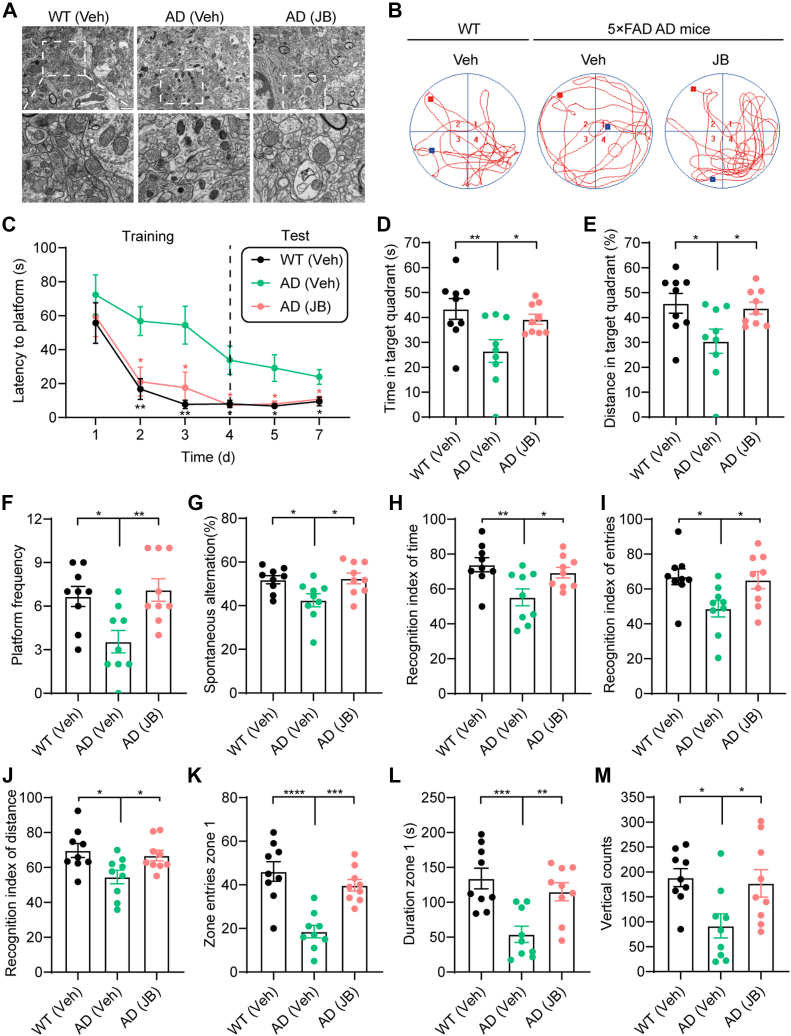
**JB restores mitophagy and improves cognitive decline in 5×FAD AD mice.***A*, electron microscopy images showing the effects of JB on mitochondrial morphology and mitophagy-like events in mouse hippocampal brain tissues. *B*, representative swimming tracks of mice on day 5 in the Morris water maze test. *C*, latency to reach the platform from day 1 to day 7 (n = 9 per group; mean ± s.e.m.; two-way ANOVA; ∗*p* < 0.0332, ∗∗*p* < 0.0021). *D*–*E*, swimming time and distance in the target quadrant on day 5. *F*, number of platform crossings on day 5. *G*, spontaneous alternation rate in Y-maze test. *H*–*J*, recognition index for time, entries and distance traveled around the novel object in the short-term novel object recognition test. *K*–*M*, open field test results: number of central zone entries, time spent in the central zone, and vertical count. n = 9 mice per group. Data are shown as mean ± s.e.m. Statistical significance was determined using one-way ANOVA. ∗*p* < 0.0332, ∗∗*p* < 0.0021, ∗∗∗*p* < 0.0002, ∗∗∗∗*p* < 0.0001. AD, Alzheimer's disease; JB, jolkinolide B.

As the common mechanisms of cancer and AD become increasingly understood, repurposing anticancer drugs represents a promising approach to identify novel therapeutic candidates for AD (13). Given that JB is a potent mitophagy inducer, we explored its potential to mitigate AD progression. Three-month-old 5×FAD AD mice and age-matched WT littermates were administered either vehicle or JB (60 mg/kg) intraperitoneally once daily for 2 months (Supplemental Fig. S8*A*). We found that JB exhibited low toxicity and no significant weight loss was observed in treated mice (Supplemental Fig. S8*B*). TEM revealed mitochondrial morphological changes in the hippocampal neurons of AD mice, characterized by reduced mitochondrial size and extensive structural damage compared with healthy WT mice (Fig. 7*A*). In addition, mitophagy defects led to substantial accumulation of damaged mitochondria in the hippocampus of AD mice, consistent with observations in AD patients (11). However, JB treatment enhanced mitophagy, facilitated the elimination of defective mitochondria, and restored normal mitochondrial morphology and size (Fig. 7*A*).

The Morris water maze test demonstrated that JB significantly improved learning and memory retention in AD mice, shortening latency time prior to reaching the platform (Fig. 7, *B* and *C*). Notably, both the time and distance spent in the target quadrant significantly increased in short-term and long-term tests after training (Fig. 7, *D* and *E*, Supplemental Fig. S8, *C* and *D*); the number of platform crossings also increased (Fig. 7*F* and Supplemental Fig. S8*E*). JB restored these parameters to levels observed in WT mice, suggesting that spatial learning and memory deficits were alleviated in 5×FAD mice after JB treatment. Furthermore, JB treatment significantly improved spatial memory performance in the Y-maze spontaneous alternation test (Fig. 7*G*). In the novel object recognition test, JB-treated AD mice exhibited increased recognition time, entry frequency, and distance traveled around new objects (Fig. 7, *H*–*J* and Supplemental Fig. S8, *F*–*H*), indicating improved cognitive function. Subsequently, we assessed the effect of JB on anxiety using an open field test. AD mice exhibited reduced entries and less time spent in the central region relative to WT mice, whereas JB treatment significantly ameliorated anxiety-like behavior (Fig. 7, *K* and *L*). In addition, JB-treated AD mice displayed increased exploratory behavior and curiosity, as reflected by a higher vertical count (Fig. 7*M*). Taken together, these findings suggest that JB, as a mitophagy inducer, improves learning, memory, and cognition function in AD mice. Thus, JB represents a potential preclinical candidate for the treatment of neurodegenerative diseases.

## Discussion

As global population aging accelerates, the incidences of chronic noncommunicable diseases, such as neurodegenerative disorders (e.g., AD) and cancer, continue to rise, such diseases are becoming the leading causes of mortality (47). However, current therapeutic approaches remain limited in terms of efficacy. Research has demonstrated that mitochondrial dysfunction is closely associated with the pathogenesis of these diseases, and the accumulation of damaged mitochondria due to dysfunction mitophagy exacerbates disease progression (48). Consequently, mitophagy-targeting approaches have emerged as promising therapeutic strategies for mitochondria-related diseases. Natural products, rich in bioactive compounds, provide a valuable resource for drug development with broad therapeutic potential. In the present study, we found that the natural diterpenoid compound JB showed robust bioactivity in pancreatic cancer and AD models, and caused almost no detectable toxic side effects in mice. In the pancreatic cancer tumor model, mechanistic studies identified mitochondrial membrane protein TOM40 as a direct target of JB, which disrupts the transport function of the TOM complex for precursor proteins. This disruption leads to increased ROS generation and compromised MMP, ultimately activating mitophagy and inducing excessive autophagic cell death in pancreatic cancer. Notably, in our AD model, JB functioned as a mitophagy activator, restoring impaired mitophagy levels and substantially mitigating learning and memory deficits. These findings highlight the dual therapeutic potential of JB in addressing both cancer and neurodegenerative diseases through modulation of mitophagy.

**Fig. 8:**
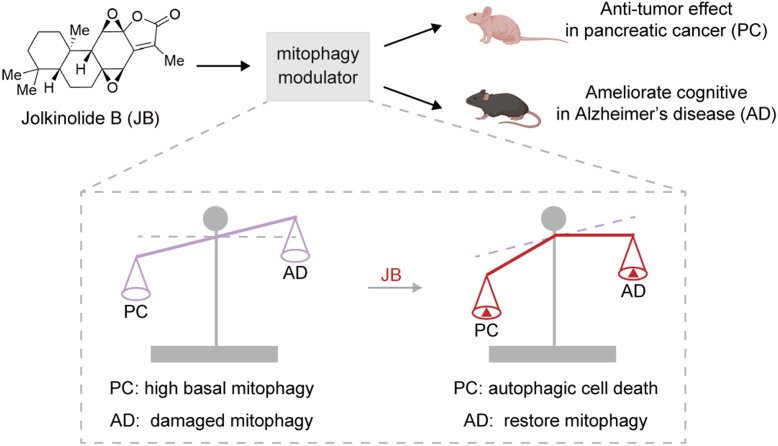
**Model diagram of JB as a mitophagy modulator in pancreatic cancer and AD.** As a mitophagy inducer, JB induces excessive autophagic cell death in PC, where basal mitophagy levels are high, thereby exerting its antitumor effect. Conversely, JB restores mitophagy in AD neurons, which exhibit impaired mitophagy, improving cognitive function. AD, Alzheimer's disease; JB, jolkinolide B; PC, pancreatic cancer.

JB, the primary active compound found in various toxic *Euphorbia* plants, has received increasing attention in recent years due to its potent and diverse pharmacological activities (29). However, its effects on pancreatic cancer and neurodegenerative diseases have not been previously reported. To our knowledge, this study is the first to demonstrate that JB exhibits significant antitumor activity against pancreatic cancer at both cellular and animal levels. Importantly, H&E staining of the heart, liver, spleen, lung, and kidney in JB-treated mice revealed no obvious morphological abnormalities, and serum levels of liver function markers (ALT and AST) and kidney function markers (UREA and CRE) showed no statistical differences from those in the vehicle group, indicating that JB did not induce obvious organ toxicity under the tested conditions. Currently, gemcitabine-based chemotherapy remains the first-line treatment for pancreatic cancer, but the enhanced efficacy of combination therapies is often accompanied by increased toxicity (49). Compared with gemcitabine, JB shows considerable promise as a therapeutic candidate for pancreatic cancer, offering a favorable balance between efficacy and toxicity. Furthermore, based on studies in both pancreatic cancer and AD disease models, we determined that JB is a potent mitophagy-targeting activator; thus, we proposed a "mitophagy modulator role" for JB (Fig. 8). In pancreatic cancer, where basal mitophagy levels are high, JB enhances mitophagy and subsequent excessive autophagic cell death (i.e., type Ⅱ programmed cell death) (50). This mechanism differs from previously reported JB-induced apoptosis in other cancers (30, 32), suggesting that the JB has distinct antitumor mechanisms according to cancer type. Conversely, in AD models where mitophagy levels are low, JB restores impaired mitophagy, normalizes mitochondrial morphology, and improves learning and memory function in behavioral assays. These findings indicate that JB may improve neuronal function by rescuing mitophagy-mediated mitochondrial homeostasis.

Although many studies have demonstrated that JB possesses broad-spectrum antitumor activity, few have investigated its direct targets, which are critical for drug discovery involving natural products. By leveraging the ability of JB to form covalent bonds with cysteine residues in target proteins, we developed a MS-based method for direct target identification without requiring structural modification of the small molecule. Through comprehensive proteomic analysis based on phenotypic changes, we identified mitochondrial protein TOM40 as a primary potential target of JB. Subsequent biochemical experiments confirmed the formation of an irreversible covalent bond between JB and TOM40-Cys74, validating the reliability of direct target identification through this MS approach. TOM40, located in the outer mitochondrial membrane, is a channel-forming subunit essential for transporting preproteins into mitochondria (42). Research has linked TOM40 to various aspects of human health and disease, including cancer, metabolic disorders, and neurodegenerative conditions (51). High TOM40 expression has been associated with poor survival outcomes in epithelial ovarian cancer and nasopharyngeal carcinoma. Studies have shown that TOM40 knockdown significantly inhibits the proliferation of epithelial ovarian cancer and nasopharyngeal carcinoma cells both *in vitro* and *in vivo* (52, 53). In this study, we found that TOM40 is highly expressed in pancreatic cancer, consistent with the active mitochondrial oxidative phosphorylation characteristic. In addition, we observed a positive correlation between JB cytotoxicity and TOM40 expression. Specifically, JB exhibited greater cytotoxicity in pancreatic and colon cancer cells than in normal pancreatic and colon epithelial cells with low TOM40 expression. Furthermore, considering the profound differences in autophagy regulatory networks, metabolic reprogramming characteristics, and signaling transduction mechanisms between tumor and normal cells (54), JB represents a highly selective and low-cytotoxicity mitophagy modulator that preferentially enhances autophagic flux in cancer cells without disrupting autophagic equilibrium in normal cells. This distinctive property offers innovative therapeutic avenues and establishes a strong theoretical framework for targeted cancer treatment strategies.

This study comprehensively elucidated the target and antitumor mechanisms of JB in pancreatic cancer, although certain limitations remain. Utilizing a combination of forward and reverse strategies in cancer models, we identified TOM40 as the primary target of JB; we confirmed its irreversible covalent binding to TOM40 through a series of *in vitro* experiments. However, other potential targets identified in this study warrant further investigation. For instance, QCR6, a subunit of the cytochrome b-c1 complex involved in the mitochondrial electron transport chain, plays a critical role in oxidative phosphorylation. Similarly, DTX1, an E3 ubiquitin-protein ligase, may participate in the ubiquitination process associated with autophagy. Further research is needed to validate the stability and functional relevance of these additional targets. In addition, although this study represents the first report of JB’s promising bioactivity in neurodegenerative disorders and provides preliminary insights regarding its mechanism for ameliorating cognitive impairment in AD mice through mitophagy restoration, some gaps remain. We observed that JB can restore mitophagy levels, but its precise molecular targets and underlying mechanisms have not been fully elucidated. Further exploration is needed concerning whether JB exerts therapeutic effects through multitarget synergies, as well as its potential synergy with other therapeutic strategies (such as anti-inflammatory and antioxidant interventions). Our ongoing research aims to comprehensively analyze the mechanism of JB through multiomics analysis, gene editing technologies, and preclinical experiments. Notably, current safety assessments of JB remain constrained to *in vitro* cellular assays and preclinical animal models. Hence, thorough toxicity profiling is essential to facilitate the clinical translation of JB. Such endeavors will not only solidify the scientific foundation for its clinical use but also may offer innovative therapeutic approaches for mitochondrial disorders.

## Conclusions

This study demonstrated the diverse biological activities of the natural compound JB against pancreatic cancer and AD, encompassing tumor growth inhibition and cognitive function enhancement. In pancreatic cancer, JB specifically disrupts the transport function of the mitochondrial target protein TOM40, thereby activating mitophagy and inducing excessive autophagic cell death, with the ultimate effect of tumor suppression. In the 5×FAD AD mouse model, JB significantly alleviates cognitive and learning deficits by restoring impaired mitophagy. Notably, JB exhibits low cytotoxicity in normal pancreatic epithelial cells and induces minimal histopathological changes in mice, as evidenced by cellular viability assays and organ pathology analyses. These findings underscore its therapeutic potential, while acknowledging that the current safety data are limited to preclinical models. Furthermore, the unique epoxy structure of JB provides a novel scaffold for covalent drug design. Overall, as a specific mitophagy activator, JB offers an innovative therapeutic strategy for mitochondria-related diseases (e.g., pancreatic cancer and AD), demonstrating substantial implications for research and clinical translation.

## Data Availability

The mass spectrometry raw data have been deposited to the ProteomeXchange Consortium (https://proteomecentral.proteomexchange.org) via the iProX partner repository with the dataset identifier PXD062393.

## Supplemental Data

This article contains supplemental data.

## Conflict of Interest

The authors declare no competing interests.

## References

[1] Nunnari J., Suomalainen A. (2012). Mitochondria: in sickness and in health. Cell.

[2] San-Millán I. (2023). The key role of mitochondrial function in health and disease. Antioxidants (Basel).

[3] Palikaras K., Lionaki E., Tavernarakis N. (2018). Mechanisms of mitophagy in cellular homeostasis, physiology and pathology. Nat. Cell Biol..

[4] Vincent A.H.J., Schulick R., Hruban R.H., Goggins M., Goggins M. (2011). Pancreatic cancer. Lancet.

[5] Yang S., Wang X., Contino G., Liesa M., Sahin E., Ying H. (2011). Pancreatic cancers require autophagy for tumor growth. Genes Dev..

[6] Chang H., Zou Z. (2020). Targeting autophagy to overcome drug resistance: further developments. J. Hematol. Oncol..

[7] Mujumdar N., Mackenzie T.N., Dudeja V., Chugh R., Antonoff M.B., Borja-Cacho D. (2010). Triptolide induces cell death in pancreatic cancer cells by apoptotic and autophagic pathways. Gastroenterology.

[8] Puissant A., Robert G., Fenouille N., Luciano F., Cassuto J.P., Raynaud S. (2010). Resveratrol promotes autophagic cell death in chronic myelogenous leukemia cells via JNK-mediated p62/SQSTM1 expression and AMPK activation. Cancer Res..

[9] EclinicalMedicine (2021). Alzheimer's disease: still in need of a cure. EClinicalMedicine.

[10] Kerr J.S., Adriaanse B.A., Greig N.H., Mattson M.P., Cader M.Z., Bohr V.A. (2017). Mitophagy and Alzheimer's disease: cellular and molecular mechanisms. Trends Neurosci..

[11] Fang E.F., Hou Y., Palikaras K., Adriaanse B.A., Kerr J.S., Yang B. (2019). Mitophagy inhibits amyloid-β and tau pathology and reverses cognitive deficits in models of Alzheimer's disease. Nat. Neurosci..

[12] Kshirsagar S., Sawant N., Morton H., Reddy A.P., Reddy P.H. (2021). Mitophagy enhancers against phosphorylated Tau-induced mitochondrial and synaptic toxicities in Alzheimer disease. Pharmacol. Res..

[13] Lee H.J., Choi H.J., Jeong Y.J., Na Y.H., Hong J.T., Han J.M. (2024). Developing theragnostics for Alzheimer's disease: insights from cancer treatment. Int. J. Biol. Macromol..

[14] Buzun K., Gornowicz A., Lesyk R., Bielawski K., Bielawska A. (2021). Autophagy modulators in cancer therapy. Int. J. Mol. Sci..

[15] Elshazly A.M., Elzahed A.A., Gewirtz D.A. (2024). The cytoprotective and cytotoxic functions of autophagy in response to mTOR inhibitors. Front. Biosci. (Landmark Ed..

[16] Spilman P., Podlutskaya N., Hart M.J., Debnath J., Gorostiza O., Bredesen D. (2010). Inhibition of mTOR by rapamycin abolishes cognitive deficits and reduces amyloid-beta levels in a mouse model of Alzheimer's disease. PLoS One.

[17] Zdanowicz A., Grosicka-Maciąg E. (2024). The interplay between autophagy and mitochondria in cancer. Int. J. Mol. Sci..

[18] Zhang L., Dai L., Li D. (2021). Mitophagy in neurological disorders. J. Neuroinflammation.

[19] Sequist L.V., Yang J.C., Yamamoto N., O’Byrne K., Hirsh V., Mok T. (2013). Phase Ⅲ study of afatinib or cisplatin plus pemetrexed in patients with metastatic lung adenocarcinoma with EGFR mutations. J. Clin. Oncol..

[20] Poels K.E., Schoenfeld A.J., Makhnin A., Tobi Y., Wang Y., Frisco-Cabanos H. (2021). Identification of optimal dosing schedules of dacomitinib and osimertinib for a phase I/Ⅱ trial in advanced EGFR-mutant non-small cell lung cancer. Nat. Commun..

[21] Witzig T.E.I.D., Inwards D. (2019). Acalabrutinib for mantle cell lymphoma. Blood.

[22] Xu W.Y.S., Zhou K., Zhou K., Pan L., Li Z., Zhou J. (2020). Treatment of relapsed/refractory chronic lymphocytic leukemia/small lymphocytic lymphoma with the BTK inhibitor zanubrutinib: phase 2, single-arm, multicenter study. J. Hematol. Oncol..

[23] Canon J.R.K., Saiki A.Y., Saiki A.Y., Mohr C., Cooke K., Bagal D. (2019). The clinical KRAS(G12C) inhibitor AMG 510 drives anti-tumour immunity. Nature.

[24] Sutanto F., Konstantinidou M., Dömling A. (2020). Covalent inhibitors: a rational approach to drug discovery. RSC. Med. Chem..

[25] Lu D., Yu X., Lin H., Cheng R., Monroy E.Y., Qi X. (2022). Applications of covalent chemistry in targeted protein degradation. Chem. Soc. Rev..

[26] Boike L.H.N., Nomura D.K., Nomura D.K. (2022). Advances in covalent drug discovery. Nat. Rev. Drug Discov..

[27] Atanasov A.G.,Z.S., Dirsch V.M., International Natural Product Sciences Taskforce, Supuran C.T., Banach M., Rollinger J.M. (2021). Natural products in drug discovery: advances and opportunities. Nat. Rev. Drug Discov..

[28] Sarwar A., Zhu M., Su Q., Zhu Z., Yang T., Chen Y. (2022). Targeting mitochondrial dysfunctions in pancreatic cancer evokes new therapeutic opportunities. Crit. Rev. Oncol. Hematol..

[29] Jian B.Z.H., Han C., Liu J., Liu J. (2018). Anti-cancer activities of diterpenoids derived from Euphorbia fischeriana Steud. Molecules.

[30] Wang Y., Shen S.Y., Liu L., Zhang X.D., Liu D.Y., Liu N. (2022). Jolkinolide B inhibits proliferation or migration and promotes apoptosis of MCF-7 or BT-474 breast cancer cells by downregulating the PI3K-Akt pathway. J. Ethnopharmacol..

[31] Gao X.H.H., Han H. (2018). Jolkinolide B inhibits glycolysis by downregulating hexokinase 2 expression through inactivating the Akt/mTOR pathway in non-small cell lung cancer cells. J. Cell Biochem..

[32] Zhang J.W.Y., Zhou Y., He Q.Y., He Q.Y. (2017). Jolkinolide B induces apoptosis of colorectal carcinoma through ROS-ER stress-Ca^2+^-mitochondria dependent pathway. Oncotarget.

[33] Pentkowski N.S., Rogge-Obando K.K., Donaldson T.N., Bouquin S.J., Clark B.J. (2021). Anxiety and Alzheimer's disease: behavioral analysis and neural basis in rodent models of Alzheimer's-related neuropathology. Neurosci. Biobehav. Rev..

[34] Xie C., Zhuang X.X., Niu Z., Ai R., Lautrup S., Zheng S. (2022). Amelioration of Alzheimer's disease pathology by mitophagy inducers identified via machine learning and a cross-species workflow. Nat. Biomed. Eng..

[35] Leger M., Quiedeville A., Bouet V., Haelewyn B., Boulouard M., Schumann-Bard P. (2013). Object recognition test in mice. Nat. Protoc..

[36] Vorhees C.V., Williams M.T. (2006). Morris water maze: procedures for assessing spatial and related forms of learning and memory. Nat. Protoc..

[37] Schenone M.D.V., Wagner B.K., Clemons P.A., Clemons P.A. (2013). Target identification and mechanism of action in chemical biology and drug discovery. Nat. Chem. Biol..

[38] Ren Y.K.A., Kinghorn A.D. (2020). Development of potential antitumor agents from the scaffolds of plant-derived terpenoid lactones. J. Med. Chem..

[39] Ziegler S.P.V., Hedberg C., Waldmann H., Waldmann H. (2013). Target identification for small bioactive molecules: finding the needle in the haystack. Angew. Chem. Int. Ed. Engl..

[40] Maertin S.E.J., Lotshaw E., Lotshaw E., Sendler M., Speakman S.D., Takakura K. (2017). Roles of autophagy and metabolism in pancreatic cancer cell adaptation to environmental challenges. Am. J. Physiol. Gastrointest. Liver Physiol..

[41] Ahting U.T.M., Engelhardt H., Hegerl R., Neupert W., Nussberger S., Nussberger S. (2001). Tom40, the pore-forming component of the protein-conducting TOM channel in the outer membrane of mitochondria. J. Cell Biol..

[42] Wiedemann N.P.N., Pfanner N. (2017). Mitochondrial machineries for protein import and assembly. Annu. Rev. Biochem..

[43] Wang Q.G.Z., Qi L., Qi L., Zhuang J., Wang C., Hong S. (2021). Structural insight into the SAM-mediated assembly of the mitochondrial TOM core complex. Science.

[44] Kowaltowski A.J., Souza-Pinto N.C., Castilho R.F., Vercesi A.E. (2009). Mitochondria and reactive oxygen species. Free Radic. Biol. Med..

[45] Lu Y.L.Z., Zhang S., Zhang T., Liu Y., Zhang L., Zhang L. (2023). Cellular mitophagy: mechanism, roles in diseases and small molecule pharmacological regulation. Theranostics.

[46] Kuzma B.A.P.I., Greenfield D.A., Ho A., Evans C.L., Evans C.L. (2021). Visualizing and quantifying antimicrobial drug distribution in tissue. Adv. Drug Deliv. Rev..

[47] Ren R.J., Huang Q., Xu G., Gu K., Dammer E.B., Wang C.F. (2022). Association between Alzheimer's disease and risk of cancer: a retrospective cohort study in Shanghai, China. Alzheimers Dement.

[48] D'Arcy M.S. (2024). Mitophagy in health and disease. Molecular mechanisms, regulatory pathways, and therapeutic implications. Apoptosis.

[49] Tempero M.A., Malafa M.P., Al-Hawary M., Behrman S.W., Benson A.B., Cardin D.B. (2021). Pancreatic adenocarcinoma, version 2.2021, NCCN clinical practice guidelines in oncology. J. Natl. Compr. Canc. Netw..

[50] Liu J.J.L.M., Yu J.Y., Liu B., Bao J.K., Bao J.K. (2011). Targeting apoptotic and autophagic pathways for cancer therapeutics. Cancer Lett..

[51] Pitt A.S.B.S., Buchanan S.K. (2021). A biochemical and structural understanding of TOM complex interactions and implications for human health and disease. Cells.

[52] Yang W.S.H., Cho H., Cho H., Chung J.Y., Lee E.J., Kim J.H. (2020). TOM40 inhibits ovarian cancer cell growth by modulating mitochondrial function including intracellular ATP and ROS levels. Cancers (Basel).

[53] Ran H.Z.J., Zeng X., Zeng X., Wang Z., Liu P., Kang C. (2023). TOM40 regulates the progression of nasopharyngeal carcinoma through ROS-mediated AKT/mTOR and p53 signaling. Discov. Oncol..

[54] Perera R.M., Bardeesy N. (2015). Pancreatic cancer metabolism: breaking it down to build it back up. Cancer Discov..

