# Supplementary material for: Jolkinolide B Activates Mitophagy to Exhibit Antipancreatic Cancer Activity and Alleviate Cognitive Deficits in Alzheimer's Disease
Source: Mol Cell Proteomics. 2025 Aug 25;24(10):101060. doi: 10.1016/j.mcpro.2025.101060 (PMC12509750; doi:10.1016/j.mcpro.2025.101060)
Supplement: Supplemental Data [file mmc7.docx]

**Jolkinolide B Activates Mitophagy to Exhibit Anti-pancreatic Cancer Activity and Alleviate Cognitive Deficits in Alzheimer's Disease**

Mingjie Gao^1^, Weiyi Hu^1^, Delan Meng^1^, Pengju Yao^1^, Siqi Yang^1^, Yuanpeng Tong^1^, Lei Wang^1^, Ya Zhang^1^, Qingsong Wang^1, *^, Jianguo Ji^1, *^, Wenyuan Zhu^1, *^

^1^*State Key Laboratory of Gene Function and Modulation Research, School of Life Sciences, Peking University, Beijing 100871, China*

**Supplemental Data**

1. Supplemental Methods

2. Supplemental Figure S1-S8

3. Supplemental Tables S1-S6

# **Supplemental Methods**

- 1. **Immunoprecipitation-Mass Spectrometry (IP-MS)**

MIA PaCa-2 cells treated with 25 μM JB or 0.1% DMSO were collected and resuspended in lysis buffer (25 mM Tris, 150 mM NaCl, 3 mM MgCl_2_, 5% glycerol, 0.5% Nonidet P-40, and 1% protease inhibitor cocktail [PI], pH 7.5) for 30 min. Lysates were centrifuged at 20,000 *g* for 30 min at 4°C; the resulting supernatants constituted protein lysates. For immunoprecipitation, 1 mg of protein was used. TOM40 antibody (Proteintech, 18409-1-AP) was added to the IP group, whereas normal rabbit IgG antibody (Cell Signaling Technology, 2729P) was added to the IgG control group. Samples were incubated with gentle rotation overnight at 4°C, followed by the addition of 30 μL Protein A-Sepharose 4B Conjugate (Invitrogen, 101041). The mixtures were incubated for an additional 12 h at 4°C, centrifuged at 2,500 rpm for 5 min at 4°C, and washed five times with wash buffer (20 mM Tris, 150 mM NaCl, 3 mM MgCl_2_, 0.2 mM ethylenediaminetetraacetic acid [EDTA], 5% glycerol, 0.1% Tween-20, pH 8.0). Samples were resuspended in wash buffer containing 4× loading buffer and heated at 100°C for 5 min. After centrifugation at 20,000 *g* for 10 min, the supernatants were collected as final elution samples for subsequent analysis.

The eluted proteins were separated via SDS–PAGE and stained with Coomassie Brilliant Blue R-250. Protein bands were excised and incubated overnight at room temperature in destaining solution (25 mM NH_4_HCO_3_/50% ACN) with rotation. The gel pieces were dehydrated with 100% ACN, incubated with 10 mM DTT/25 mM NH_4_HCO_3_ for 1 h at 56°C, and then incubated with 55 mM IAA/25 mM NH_4_HCO_3_ for 1 h at room temperature. Gel pieces subsequently were digested overnight with trypsin at 37°C. Peptides were extracted twice with ACN containing 0.1% formic acid, and vacuum-dried for mass spectrometry analysis.

- 1. **Construction of TOM40-Knockdown MIA PaCa-2 Cells (shTOM40)**

To generate MIA PaCa-2 cells with stable knockdown of TOM40, the human TOM40 shRNA sequence 5'-CATGTCTCTAGCTGGGAAATA-3' was inserted into the pLKO.1 vector. The pLKO.1 empty vector was used to create negative control cells (shNC). Plasmids PLP1, PLP2, VSVG, and either pLKO.1-shTOM40 or pLKO.1-shNC were co-transfected into 293T cells using the transfection reagent polyethylenimine (PEI; EarthOx, 23966). Lentiviral particles were collected from the 293T cell medium 48 h after transfection and used to infect MIA PaCa-2 cells. Infected cells were selected using 1 μg/mL puromycin (InvivoGen, ant-pr-1) and sorted into single, viable cells in 96-well plates using flow cytometry (BD Aria Fusion). Monoclonal cells were expanded, and TOM40 expression was analyzed by Western blotting to identify positive clones for subsequent experiments.

- 1.
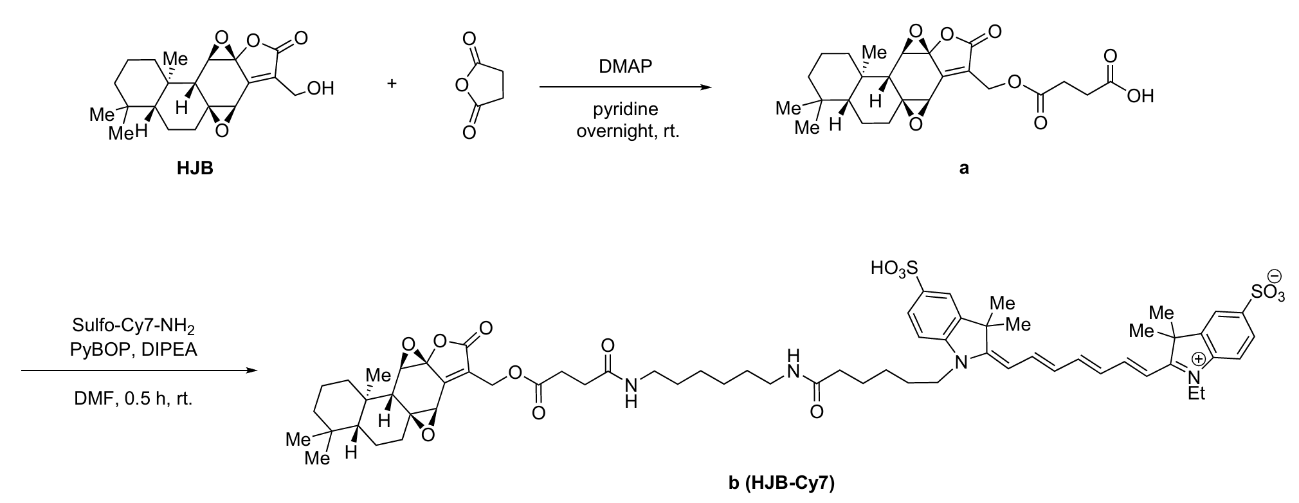
 **Synthesis of HJB-Cy7**

HJB (15 mg, 43.3 μmol, 1 equiv.), succinic anhydride (21.67 mg, 5.0 equiv.) and DMAP (2.64 mg, 0.5 equiv.) were dissolved in 1 mL anhydrous pyridine. The mixture was stirred at room temperature overnight, monitored by thin-layer chromatography (TLC). Until completion of the reaction, the solution was concentrated under reduced pressure. Subsequently, 15 mL dichloromethane was added, and the mixture was sequentially washed with 10% citric acid solution and saturated brine, followed by drying over Na_2_SO_4_. The mixture was then filtered, and the filtrate was concentrated under reduced pressure to afford the intermediate product (**a**).

The residue was redissolved in 1 mL DMF. Then Sulfo-Cy7-NH_2_ (30.44 mg, 0.9 equiv.), PyBOP (29.29 mg, 1.3 equiv.), and DIPEA (27.98 mg, 5.0 equiv.) were added to the reaction mixture. The mixture was stirred at room temperature for 30 min to ensure complete reaction. Then mixture was concentrated under reduced pressure. The residue was redissolved in 1 mL ethanol and precipitated by the addition of 100 mL cold diethyl ether. The product was collected by filtration and washed with cold diethyl ether to yield the desired product of HJB-Cy7 (**b**).

The product was white solid, 10 mg, 21% yield over two steps. ^1^H NMR (400 MHz, DMSO-*d*_6_) δ 8.16 (s,1H), 7.95 – 7.77 (m, 3H), 7.73 (t, *J* = 2.0 Hz, 3H), 7.69 (t, *J* = 5.6 Hz, 1H), 7.62 (m, 2H), 7.28 (t, *J* = 7.9 Hz, 2H), 6.53 (t, *J* = 12.6 Hz, 2H), 6.36 (dd, *J* = 13.7, 6.4 Hz, 2H), 4.96 (s, 2H), 4.24 (s, 1H), 4.17 (s, 1H), 4.15 – 4.08 (m, 2H), 4.07 – 4.00 (m, 2H), 2.98 (m, 4H), 2.53 (m, 3H), 2.37 – 2.31 (m, 3H), 2.02 (t, *J* = 7.3 Hz, 2H), 1.97 – 1.83 (m, 2H), 1.69 (m, 3H), 1.62 (s, 15H), 1.56 – 1.48 (m, 3H), 1.44 (m, 4H), 1.33 (m, 8H), 1.09 (s, 3H), 0.90 - 0.84 (m, 4H), 0.79 (s, 3H), 0.69 (s, 3H). MS (ESI): calcd. for C_65_H_84_N_4_O_14_S_2_ [M+H]^+^: 1209.5498; found: 1209.5564.


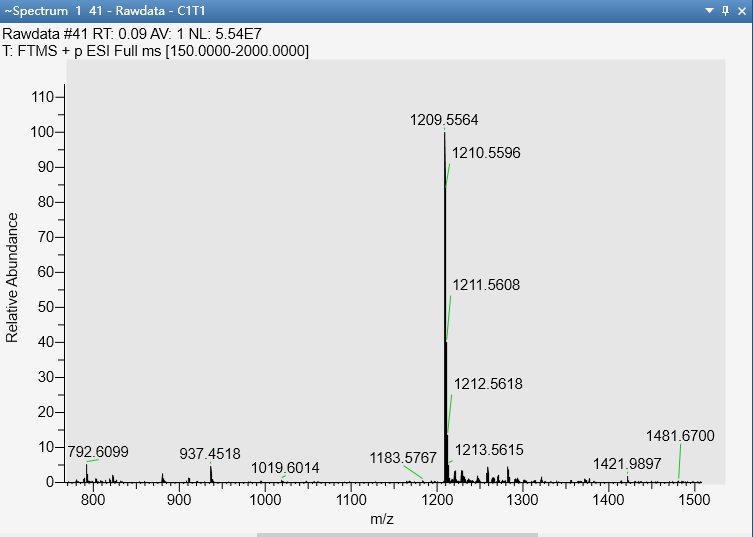

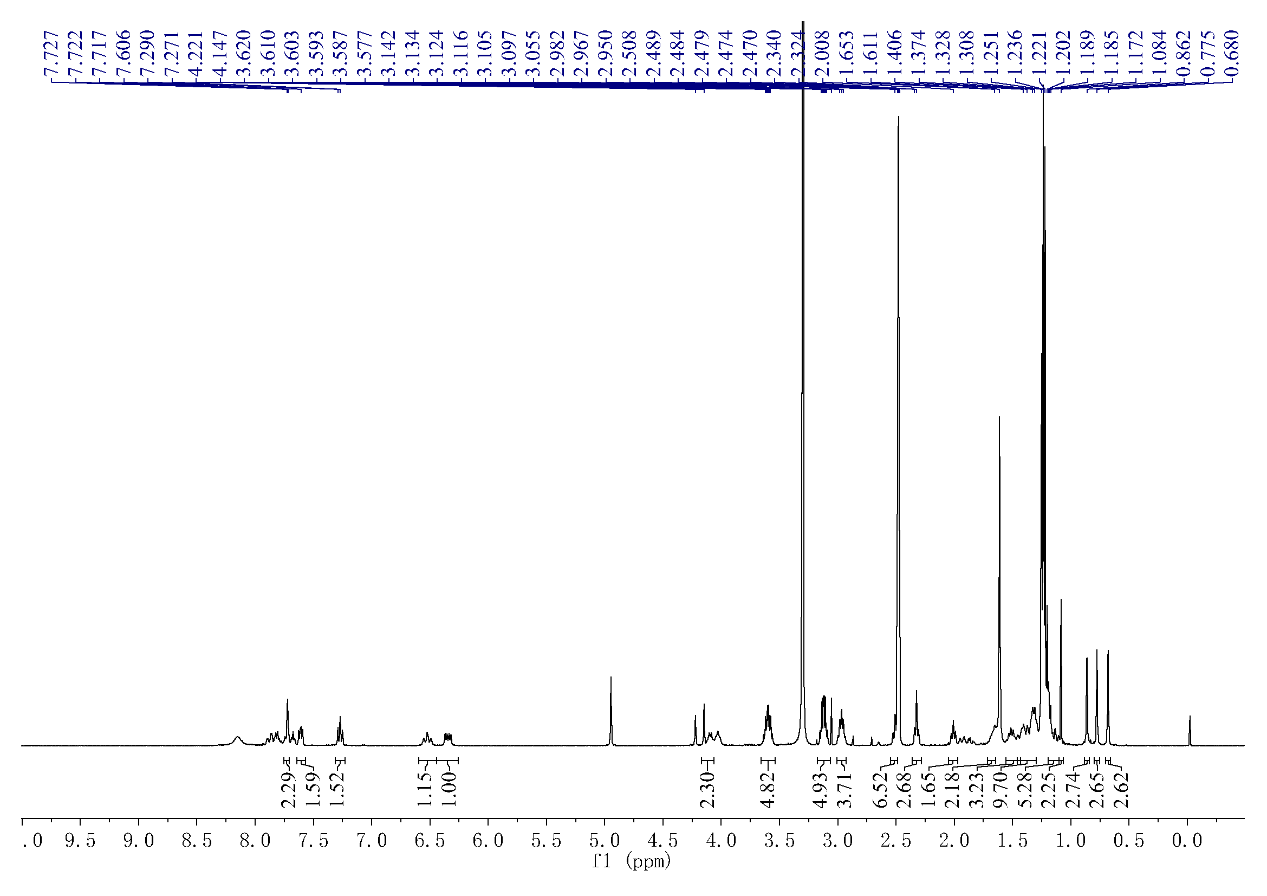
^1^H NMR of HJB-Cy7

MS of HJB-Cy7

- 1. **Serum Biochemical Analysis**

Blood samples were collected as previously described, and serum was separated by centrifugation for subsequent assessment of liver and kidney function markers. All biochemical analyses were performed using commercial kits from Meikang Biotech Co., Ltd. (Ningbo, China) in strict accordance with the manufacturer’s protocols. Liver function was evaluated by measuring alanine aminotransferase (ALT) activity via the alanine substrate method (H001), with enzymatic activity quantified by monitoring NADH consumption at 340 nm, while aspartate aminotransferase (AST) was assessed using the aspartate substrate method (H002), calculating activity based on NADH depletion at the same wavelength. Kidney function was analyzed by determining urea (UREA) concentration through the urease-glutamate dehydrogenase method (H105), with NADH oxidation rates at 340 nm calibrated against standard curves, and creatinine (CRE) levels were measured via the sarcosine oxidase method (H106), where absorbance changes at 546 nm (ΔA) exhibited a linear correlation with CRE concentration. All assays were conducted on a Sysmex BX-3010 automated biochemical analyzer to ensure standardized detection.

# **
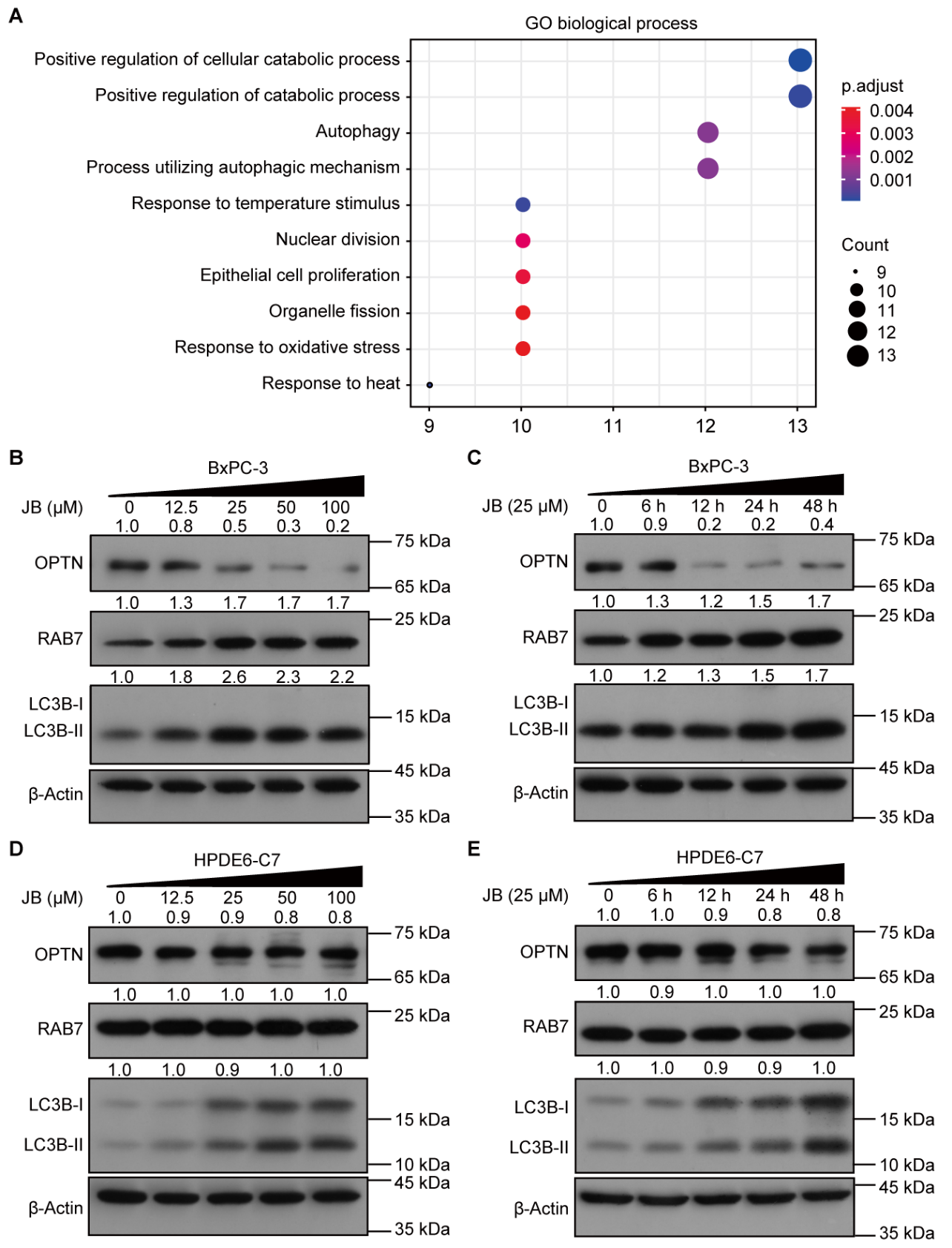
Supplemental Figures**

**Fig. S1. JB induces increased mitophagy in pancreatic cancer cells but not in normal cells.** (A) Biological process enrichment analysis of significantly differentially expressed proteins. (B, C) Pancreatic cancer BxPC-3 cells were treated with the indicated concentrations of JB for 24 h or with 25 μM JB for 0, 6, 12, 24, and 48 h. Whole-cell lysates were collected and analyzed by Western blotting. (D, E) Human normal pancreatic epithelial HPDE6-C7 cells were treated with JB (0, 12.5, 25, 50, 100 μM) for 24 h or with 25 μM JB for the indicated durations. Whole-cell lysates were collected and analyzed by Western blotting.

**
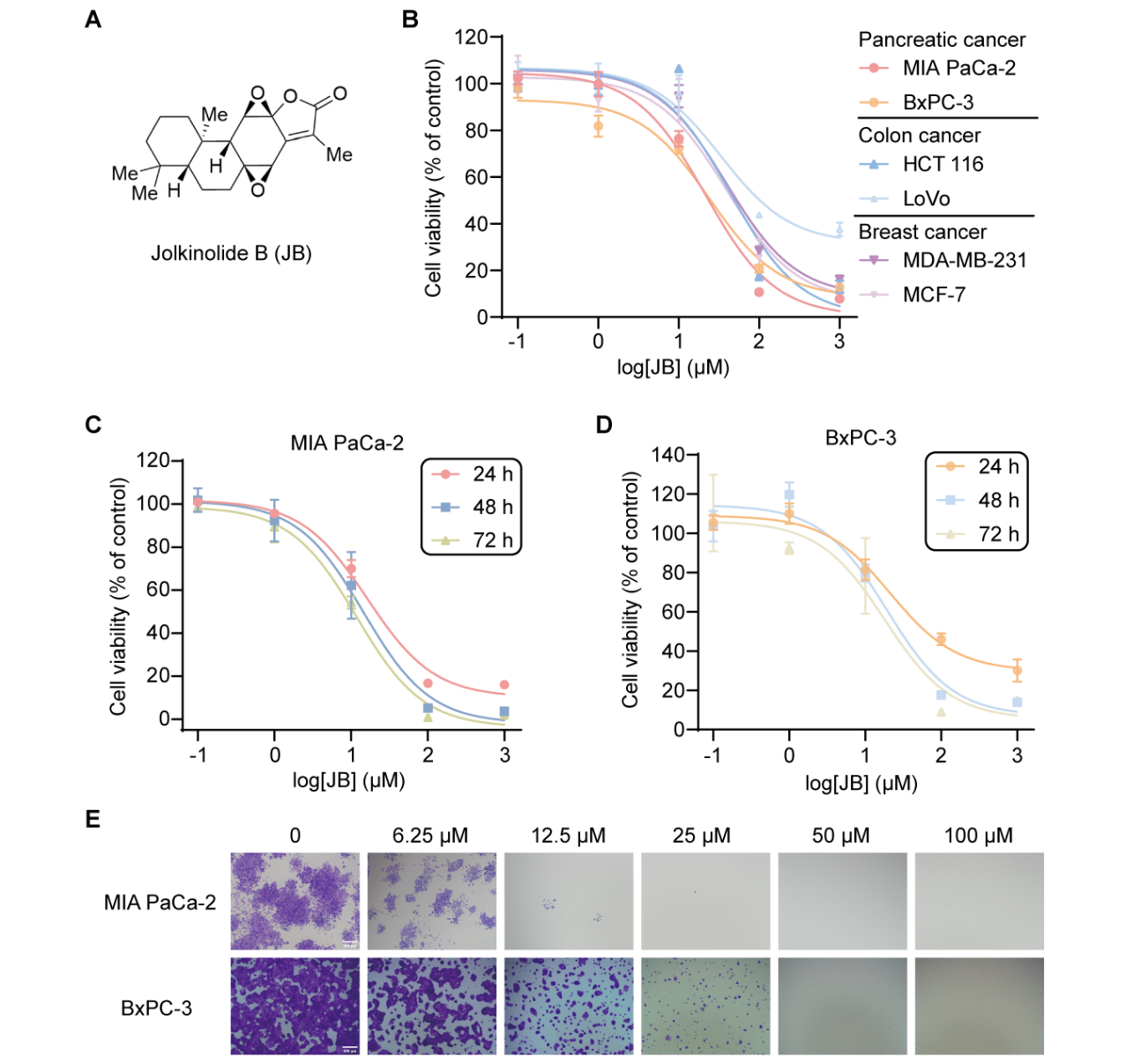
Fig. S2. JB inhibits the growth and proliferation of pancreatic cancer cells.** (A) Chemical structure of Jolkinolide B (JB). (B) Viabilities of MIA PaCa-2, BxPC-3, HCT 116, LoVo, MDA-MB-231, and MCF-7 cell lines treated with the indicated concentrations of JB for 24 h, as determined by CCK-8 assay. (C, D) MIA PaCa-2 and BxPC-3 cells were treated with the indicated concentrations of JB, and cell viability was measured using the CCK-8 assay at 24, 48, and 72 h. Data are presented as mean ± s.e.m. from three independent experiments. (E) MIA PaCa-2 and BxPC-3 cells were treated with JB (0, 6.25, 12.5, 25, 50, 100 μM) for 5 days and stained with crystal violet solution to visualize colonies. Scale bar: 500 μm.

**
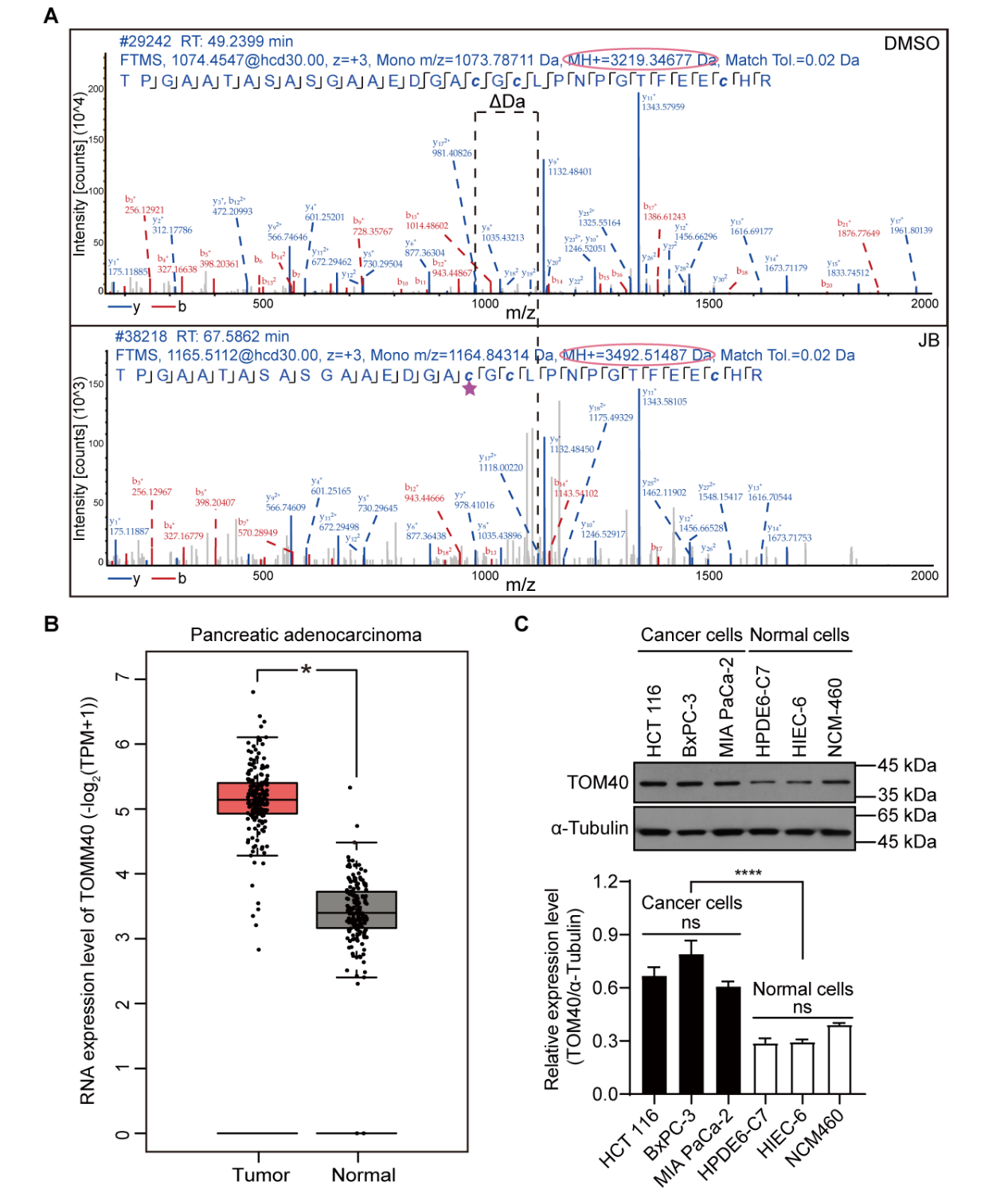
Fig. S3. The potential target protein TOM40 is highly expressed in pancreatic cancer.** (A) Secondary spectrum of a JB-modified peptide from the potential target TOM40. The spectrum for the DMSO control group is shown at the top, and the spectrum for the JB treatment group is shown at the bottom. The red circle highlights the actual parent ion mass of the corresponding peptide; the purple star indicates the JB modification site. (B) RNA expression levels of *TOMM40* were obtained from the TCGA and GTEx databases and plotted using the GEPIA web server. Statistical analysis included 179 tumor tissues from pancreatic cancer patients and 171 paired normal tissues. TCGA: The Cancer Genome Atlas; GTEx: Genotype-Tissue Expression Project. (C) Analysis was performed to detect TOM40 protein expression in cancer and normal cells. α-Tubulin was used to normalize the relative expression levels of TOM40. Data are presented as mean ± s.e.m. from three independent experiments. Statistical significance was determined using two-tailed unpaired Student’s *t*-test; *****P* < 0.0001.

**
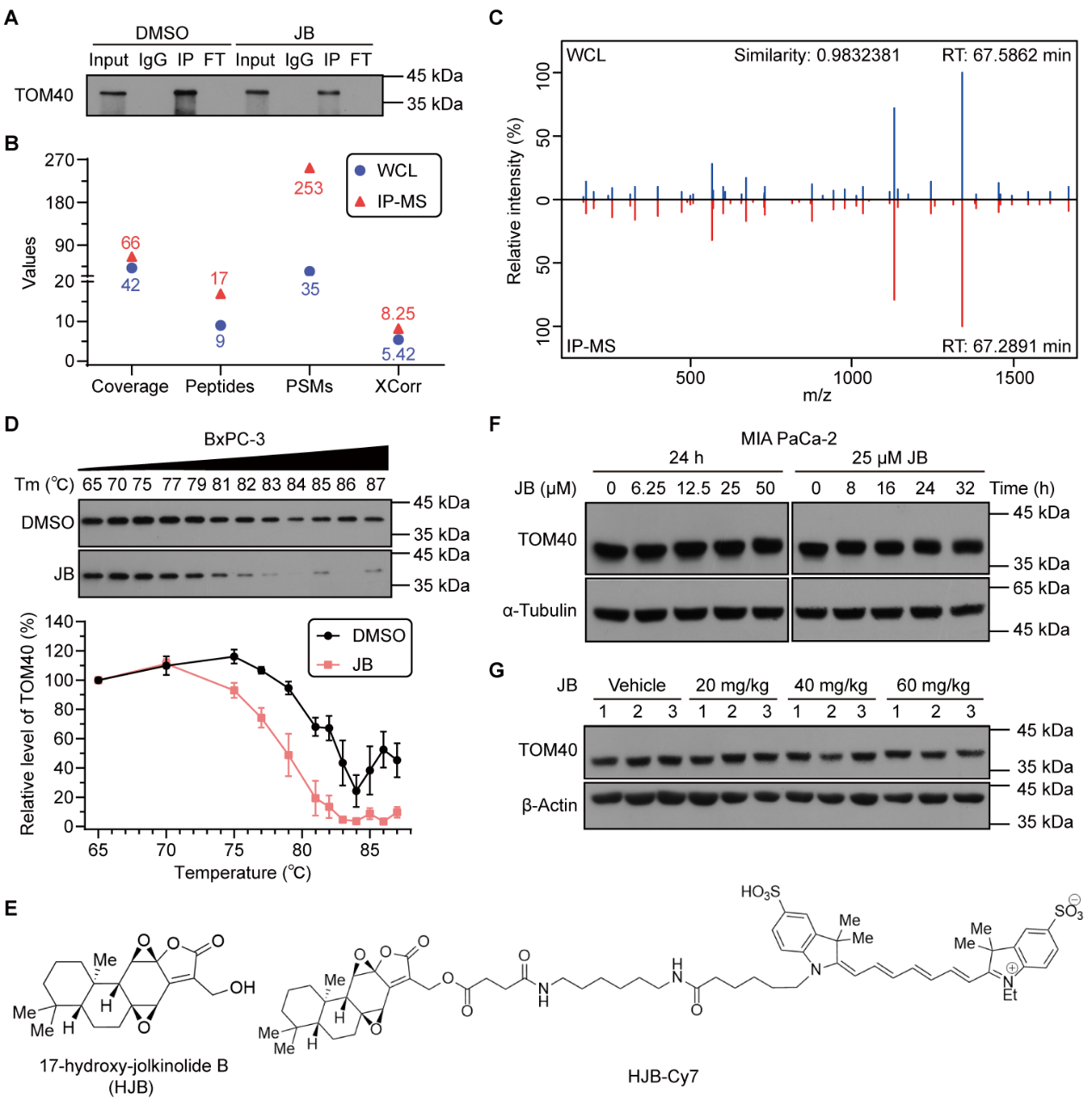
Fig. S4. JB covalently binds to the target TOM40 in pancreatic cancer.** (A-C) Immunoprecipitation was used to enrich TOM40 protein, and mass spectrometry was conducted to identify JB modifications in MIA PaCa-2 cells. Western blotting was performed to confirm protein enrichment (A). Mass spectrometry results are shown for WCL and IP-MS samples (B). Spectral similarity analysis and visualization are presented (C). Input: whole-cell lysates; IP: TOM40 antibody enrichment; IgG: normal rabbit IgG antibody enrichment; FT: flow-through; WCL: whole-cell lysate; IP-MS: immunoprecipitation–mass spectrometry-enriched group; PSMs: peptide spectrum matches; RT: retention time. (D) CETSA was used to measure the binding affinity of JB to TOM40 in pancreatic cancer BxPC-3 cells. Data are presented as mean ± s.e.m. (n = 3). (E) Chemical structures of 17-hydroxy-jolkinolide B (HJB) and the synthesized probe HJB-Cy7. (F, G) Western blotting analysis was performed to detect TOM40 expression levels and the mass shift resulting from the interaction between JB and TOM40 in cells (F) and in mice (G).

**
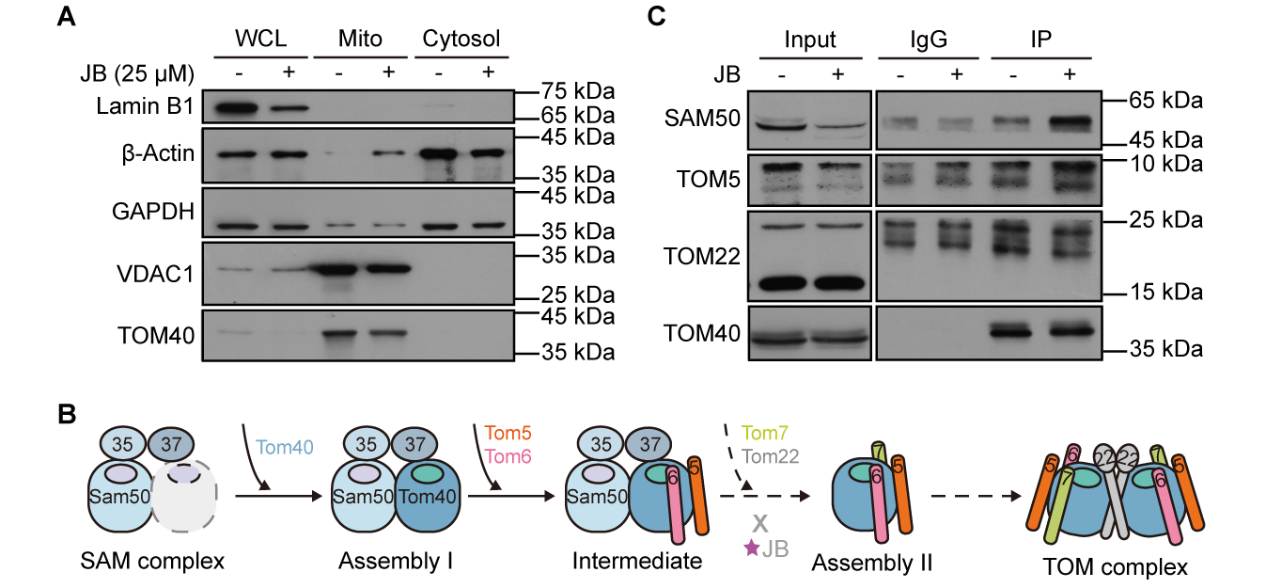
Fig. S5. Isolation of high-purity mitochondria and disruption of TOM complex assembly.** (A) Western blotting analysis was performed to confirm mitochondrial purity using cell localization markers: Lamin B1 as a nuclear marker, β-Actin and GAPDH as cytoplasmic markers, and VDAC1 and TOM40 as mitochondrial markers. (B) Schematic diagram of the TOM complex assembly process. (C) MIA PaCa-2 cells were treated with 25 μM JB or DMSO for 24 h. Co-immunoprecipitation assays were performed to enrich proteins interacting with TOM40, followed by quantification via Western blotting.

**
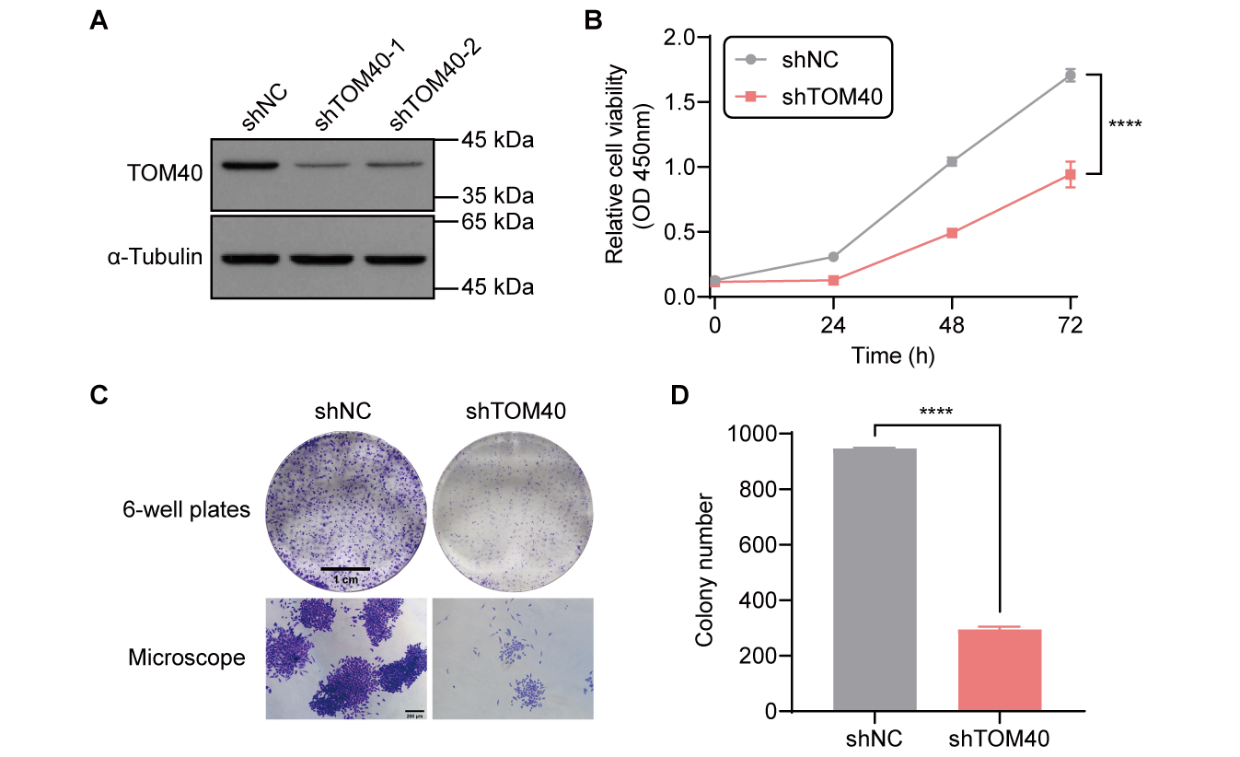
Fig. S6. TOM40 knockdown inhibits the proliferation of pancreatic cancer MIA PaCa-2 cells.** (A) The TOM40-knockdown MIA PaCa-2 cell line (shTOM40) was generated, and Western blotting analysis was performed to confirm TOM40 expression levels in negative control cells (shNC) and shTOM40 cells. (B) Cell viabilities of shNC and shTOM40 cells were assessed using the CCK-8 assay. Data are shown as mean ± s.e.m. (n = 4). Statistical significance was determined by two-way ANOVA, *****P* < 0.0001. (C, D) Colony survival assays were conducted to evaluate the long-term proliferation capacity of shNC and shTOM40 cells. Colony numbers were quantified using ImageJ. Scale bars: 1 cm for the 6-well plate images and 200 μm for the microscopic images. For D, data are shown as mean ± s.e.m. from three independent experiments. Statistical significance was determined using two-tailed unpaired Student’s *t*-test; *****P* < 0.0001 compared with the control group.

**
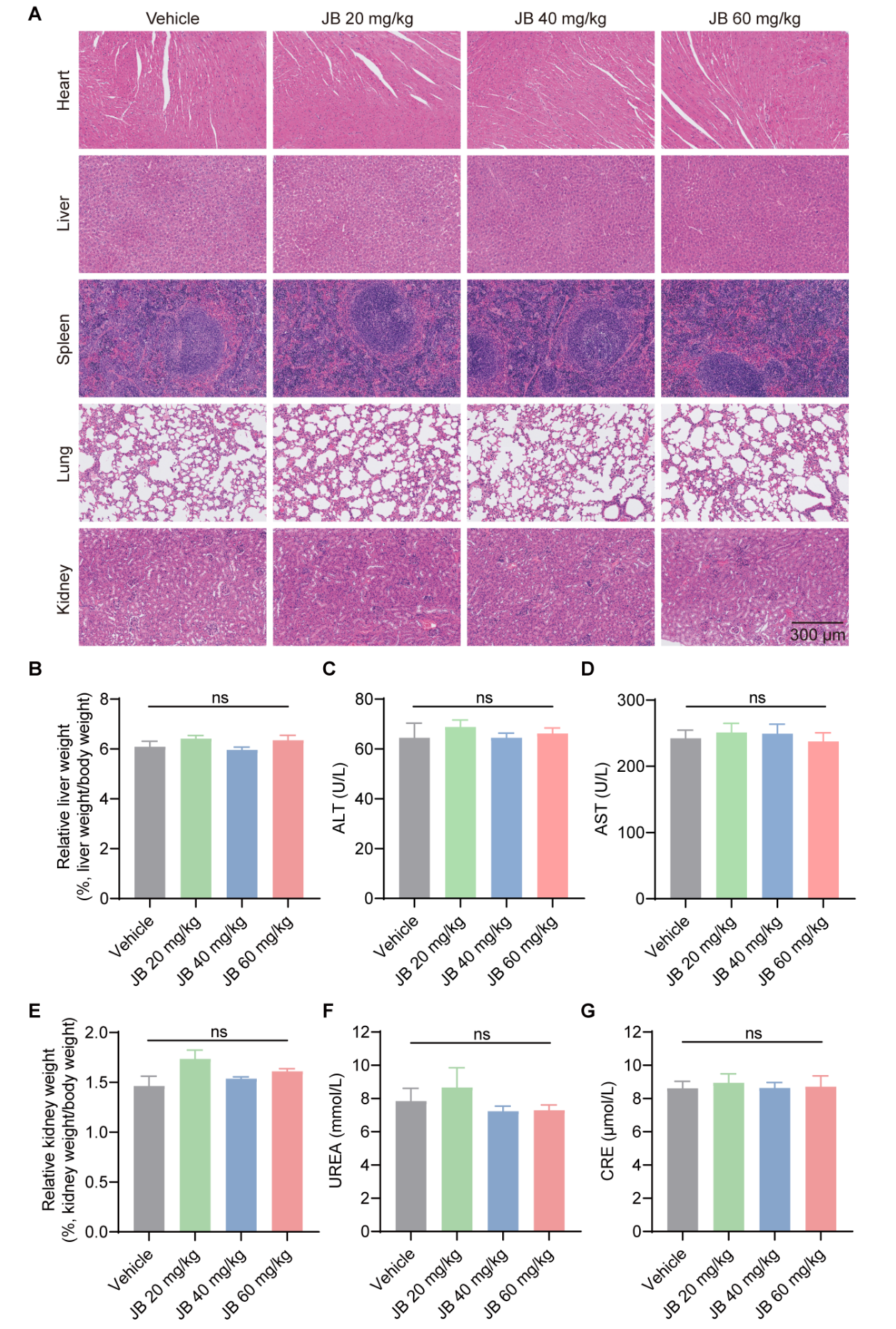
Fig. S7. JB does not exhibit obvious toxic effects on tumor-bearing mice.** (A) Representative images of H&E staining of the heart, liver, spleen, lung, and kidney. (B) Liver weights were measured after dissection. (C, D) Blood was collected via retro-orbital bleeding for serum biochemical analysis. Levels of alanine transaminase (ALT) and aspartate transaminase (AST) were measured to assess liver function. (E) Kidney weights were measured after dissection. (F, G) Blood urea nitrogen (UREA) and creatinine (CRE) levels were measured to evaluate kidney function. Data are presented as mean ± s.e.m. (n = 6 independent mice). Statistical significance was determined by one-way ANOVA. ns, not significant.

**
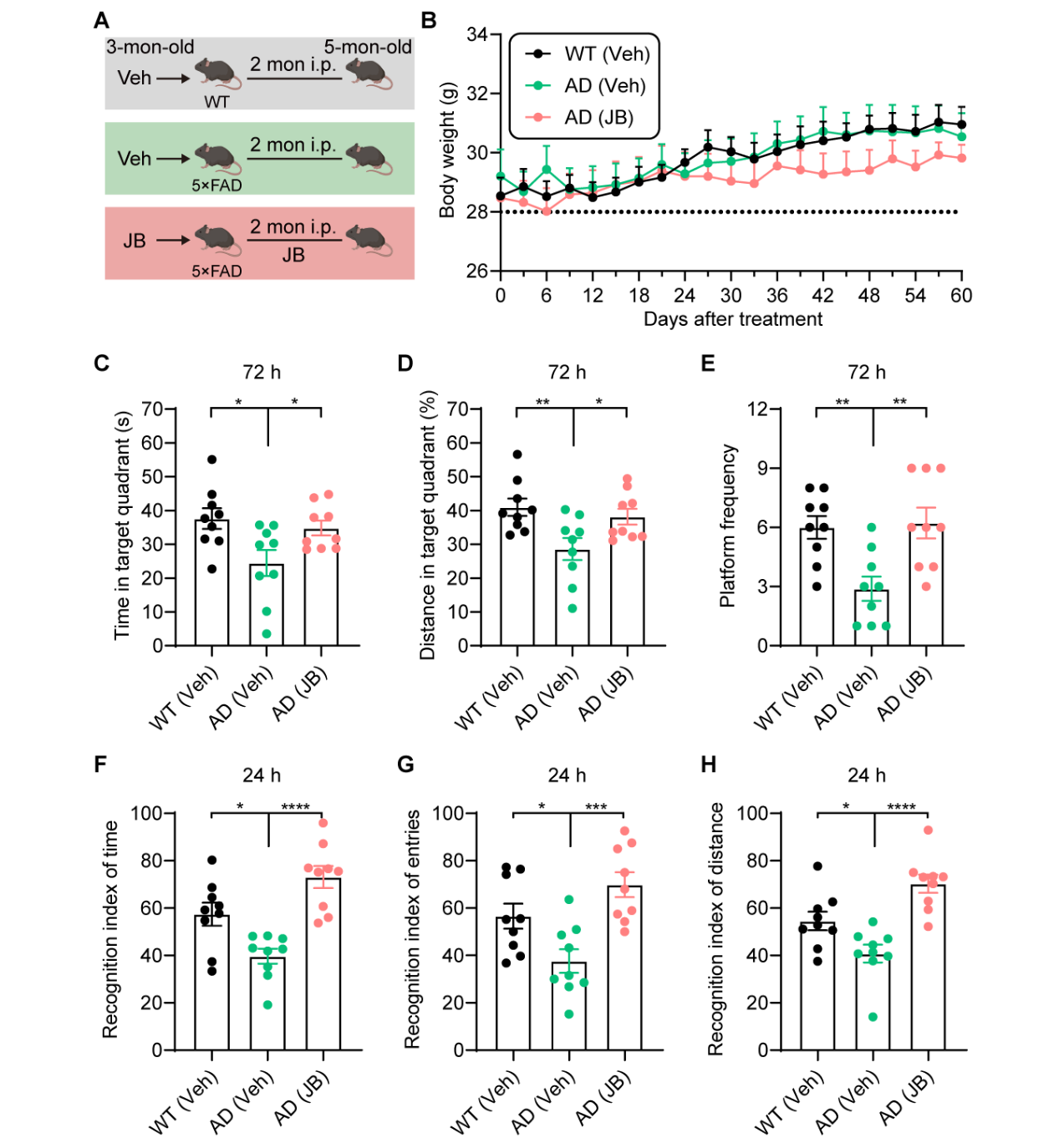
Fig. S8. JB improves long-term memory and cognitive function in AD mice with minimal side effects.** (A) Beginning at 3 months of age, 5×FAD AD mice and WT mice were administered 60 mg/kg JB or vehicle daily via intraperitoneal injection for 2 months, then subjected to behavioral testing and tissue collection. (B) Body weight was measured every 2 days and recorded over the 2-month treatment period. (C, D) Swimming time and distance in the target quadrant on day 7 of the Morris water maze test. (E) Number of platform crossings on day 7. (F-H) Recognition index for time, entries, and distance traveled around the novel object in the long-term novel object recognition test. Data are shown as mean ± s.e.m. (n = 9 mice per group). Statistical significance was determined by one-way ANOVA. **P*< 0.0332, ***P* < 0.0021, ****P* < 0.0002, *****P* < 0.0001.

# **Supplemental tables**

**Table S1. Antibodies for Western blotting.**

**Table S2. Quantitative proteomic data of MIA PaCa-2 cells.**

**Table S3. Cellular assay data.**

**Table S4. The m/z and intensity of b ions and y ions from modified peptide of TOM40.**

**Table S5. Quantitative proteomic data of high-purity mitochondria.**

**Table S6. Proteins identified based on single unique peptide.**
