# Supplementary material for: Jolkinolide B Activates Mitophagy to Exhibit Antipancreatic Cancer Activity and Alleviate Cognitive Deficits in Alzheimer's Disease
Source: Mol Cell Proteomics. 2025 Aug 25;24(10):101060. doi: 10.1016/j.mcpro.2025.101060 (PMC12509750; doi:10.1016/j.mcpro.2025.101060)
Supplement: Supplemental Material [file mmc8.docx]

**Annotated spectra for proteins with single unique peptide**

| **Dateset** | **Accession** | **Peptide** | **Spectar number** |
| --- | --- | --- | --- |
| **Table S2** | **P55061 [8-15]** | **[K].INFDALLK.[F]** | **2** |

**
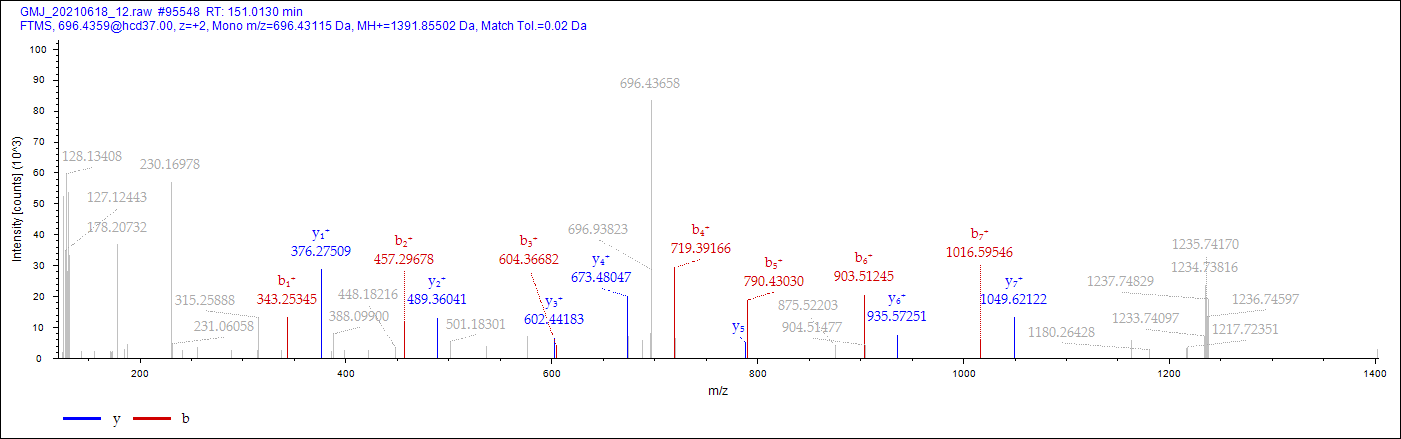
**

**
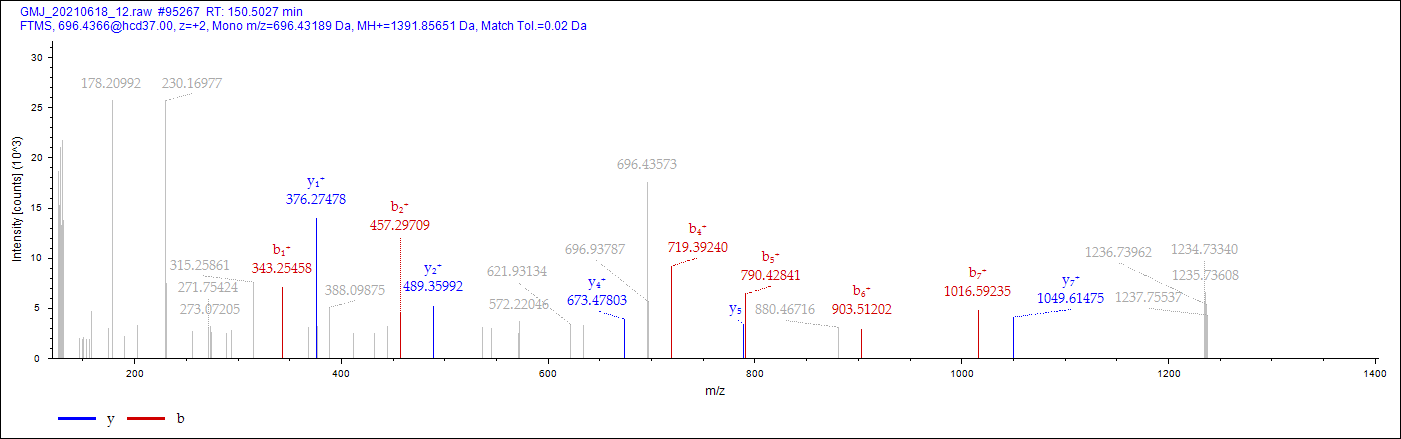
**

| **Dateset** | **Accession** | **Peptide** | **Spectar number** |
| --- | --- | --- | --- |
| **Table S2** | **Q8NEP3 [375-380]** | **[K].MELFVK.[E]** | **5** |

**
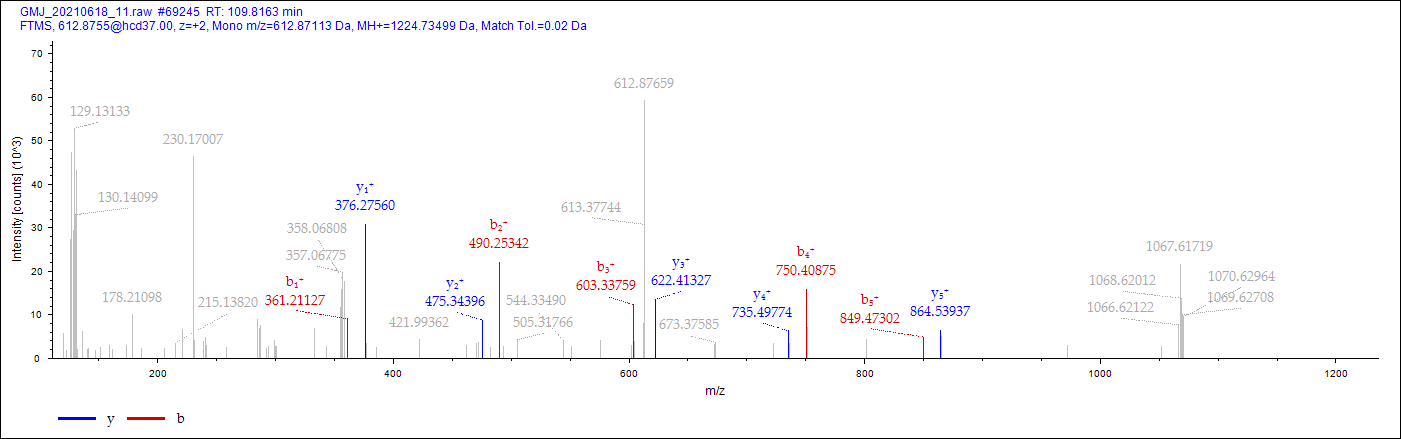

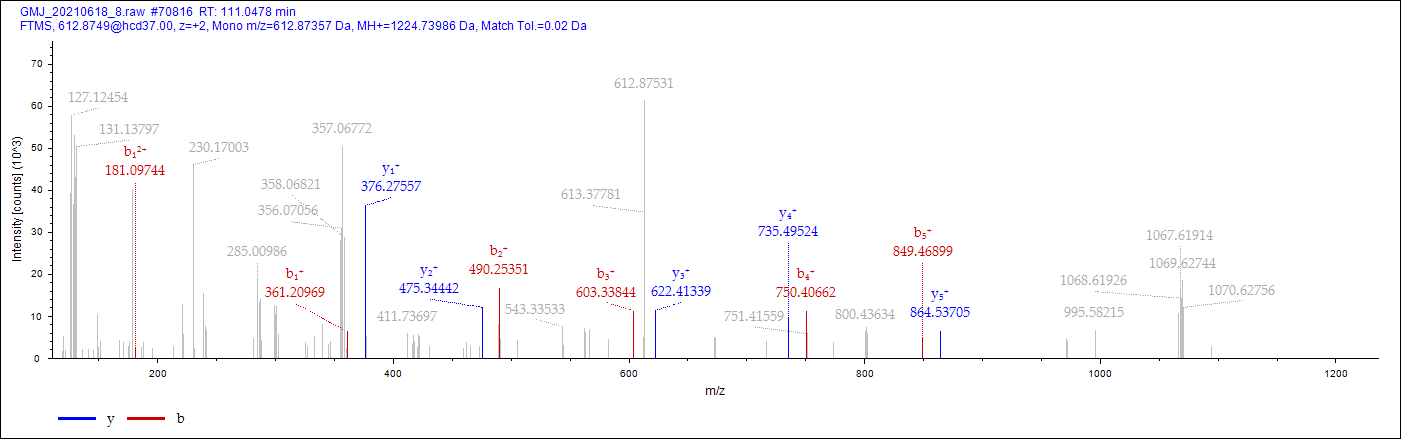

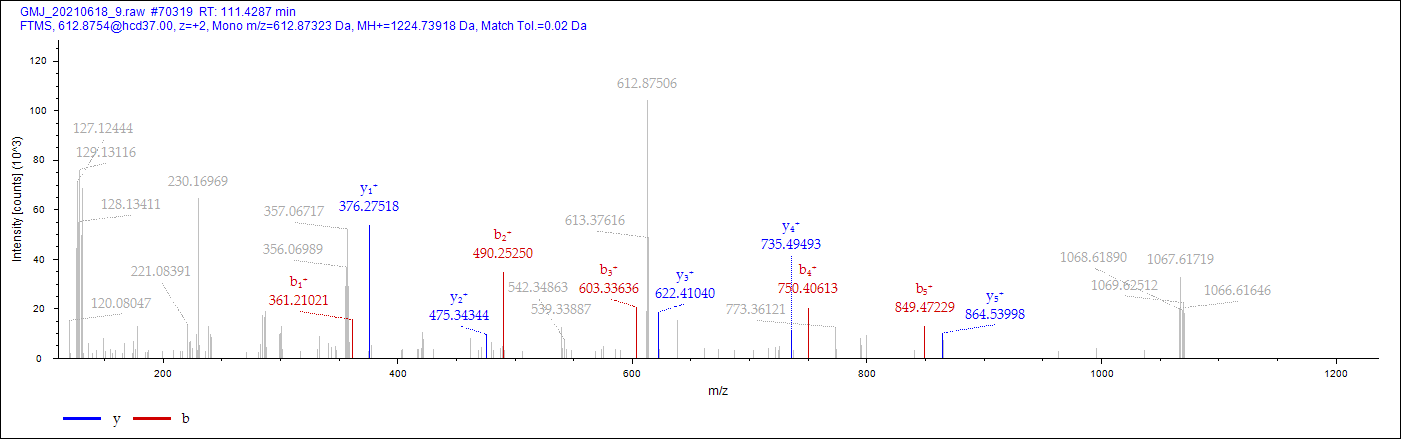

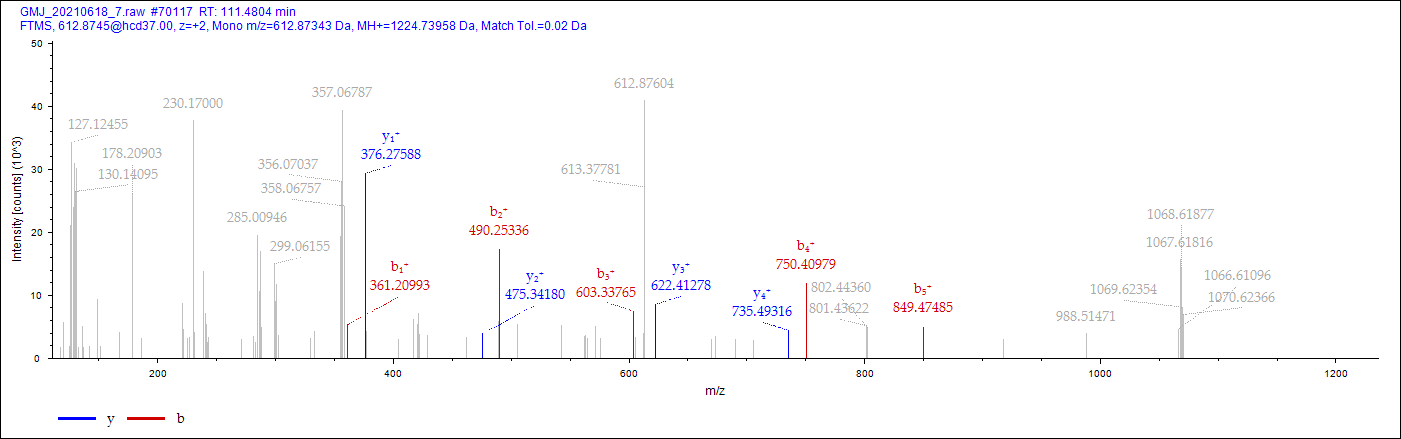

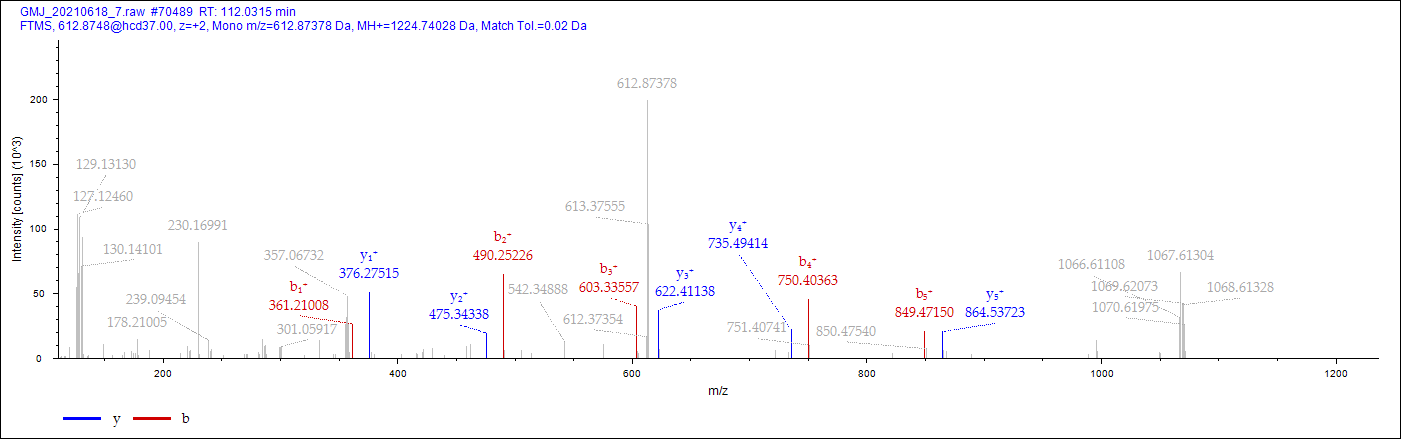
**

| **Dateset** | **Accession** | **Peptide** | **Spectar number** |
| --- | --- | --- | --- |
| **Table S2** | **P30408 [37-45]** | **[K].YASENHLSR.[F]** | **9** |

**
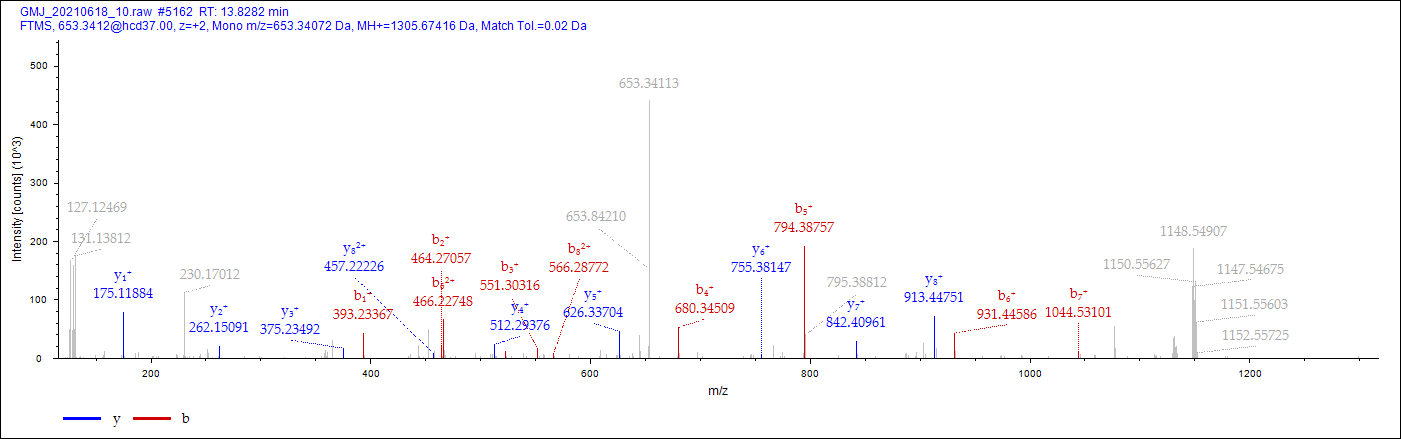

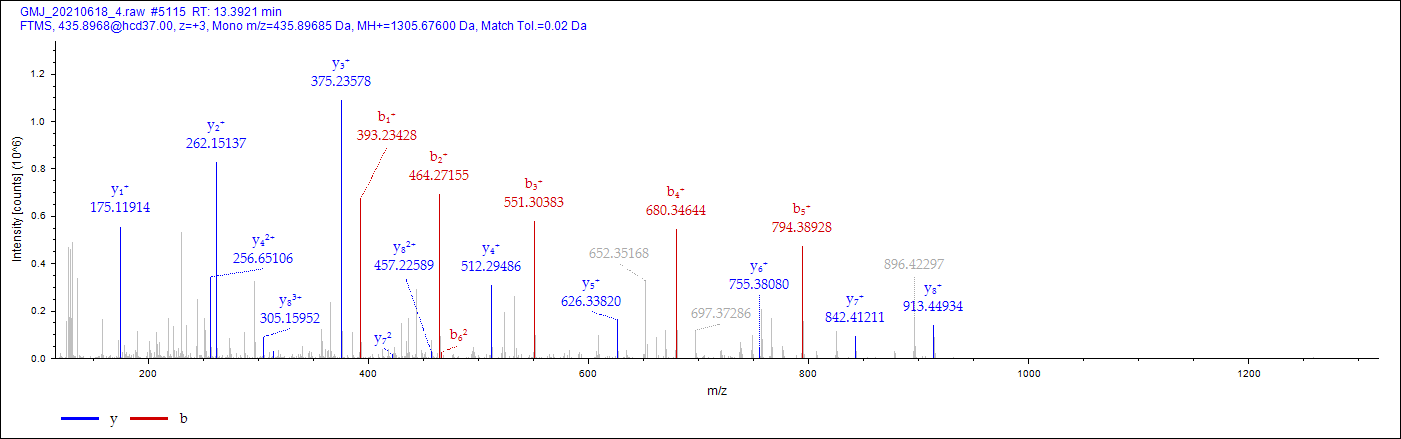

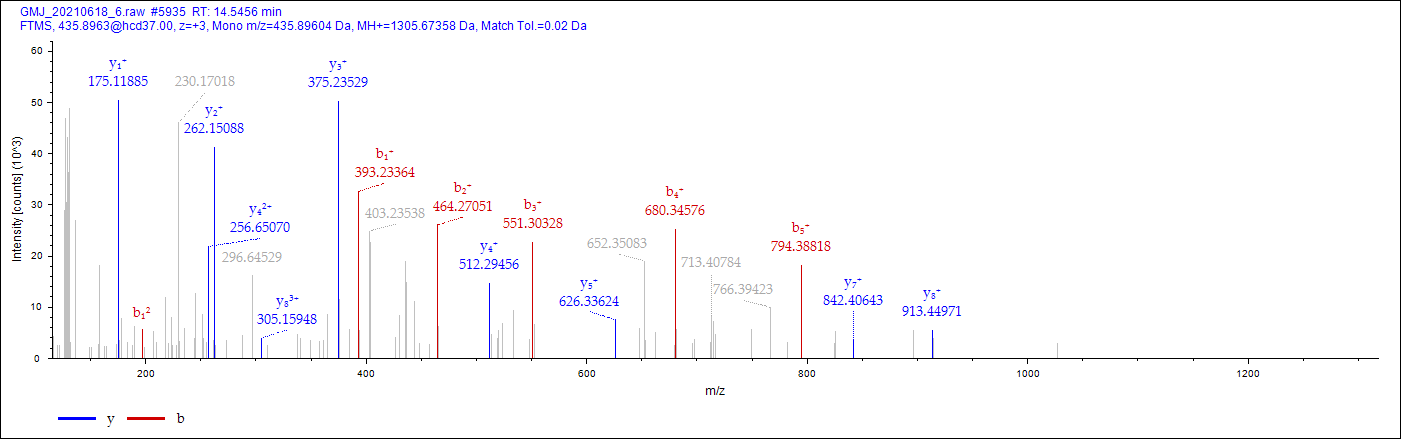

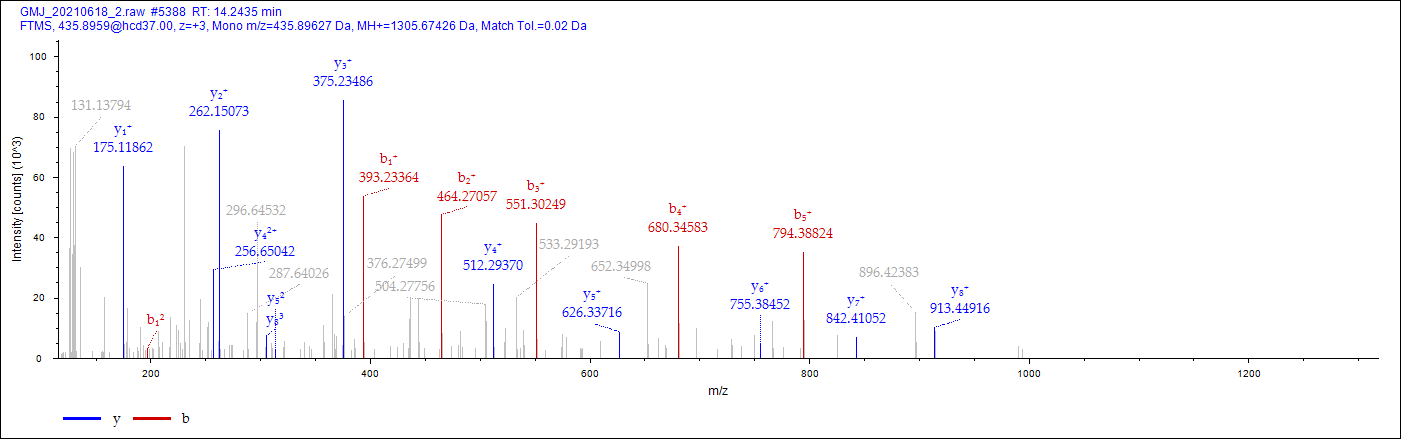

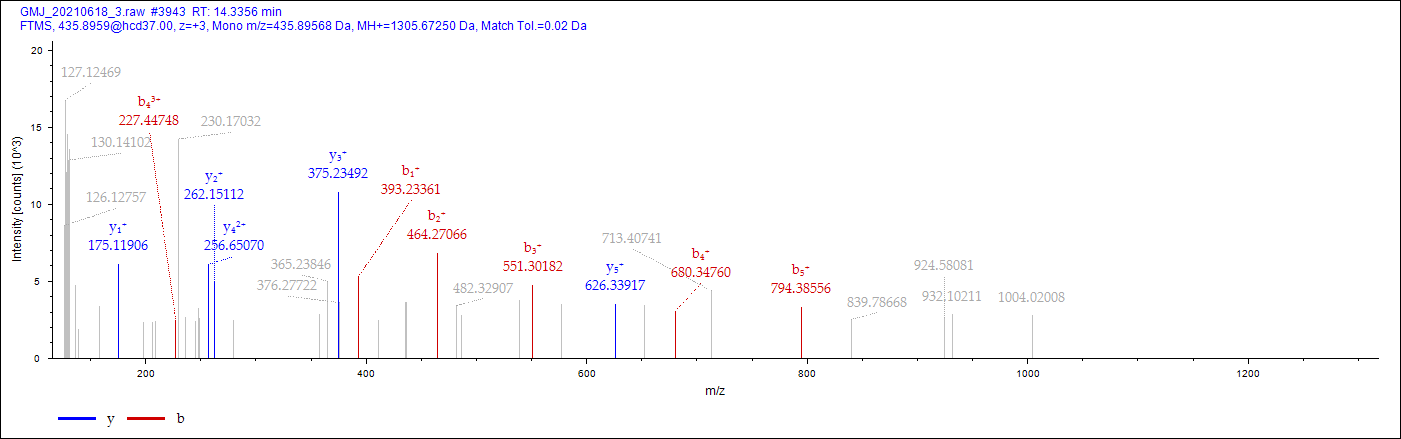

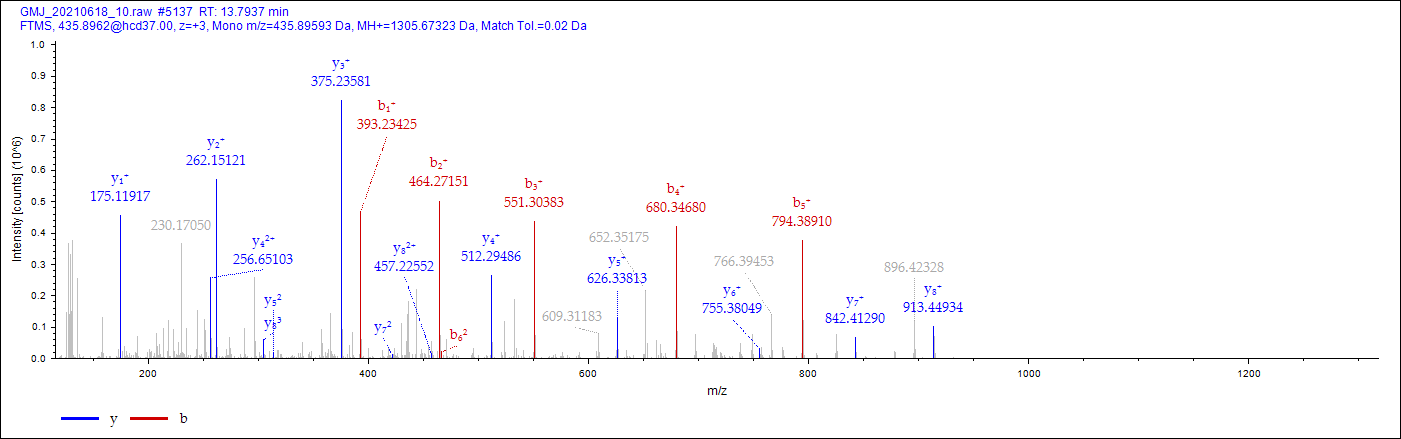

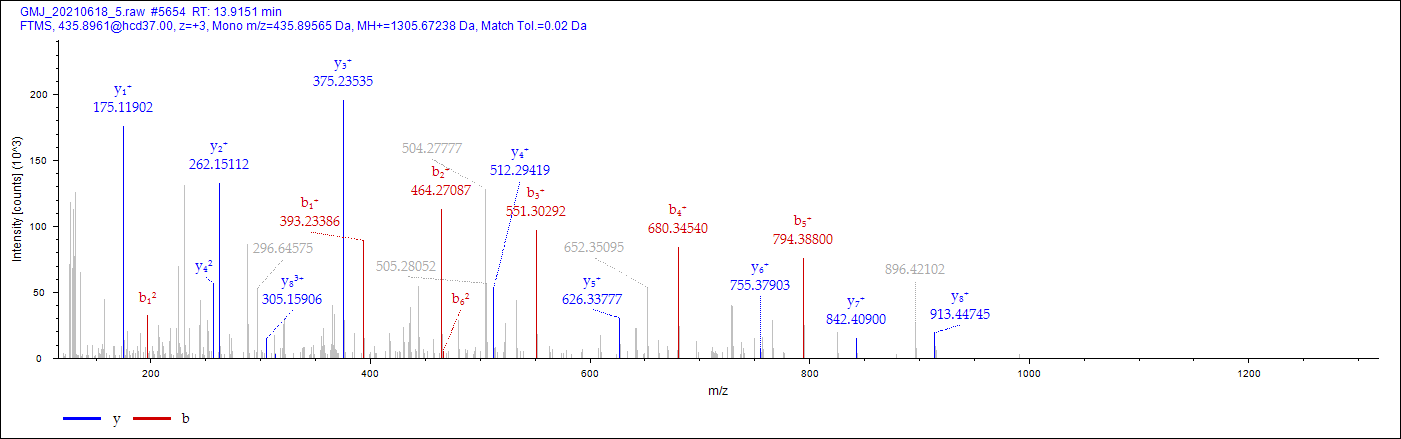

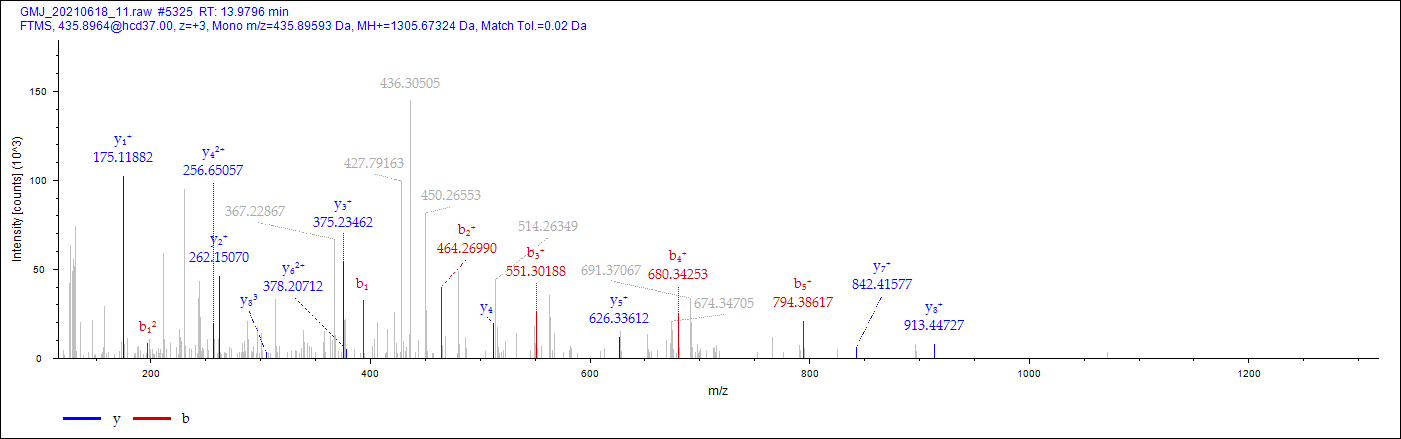

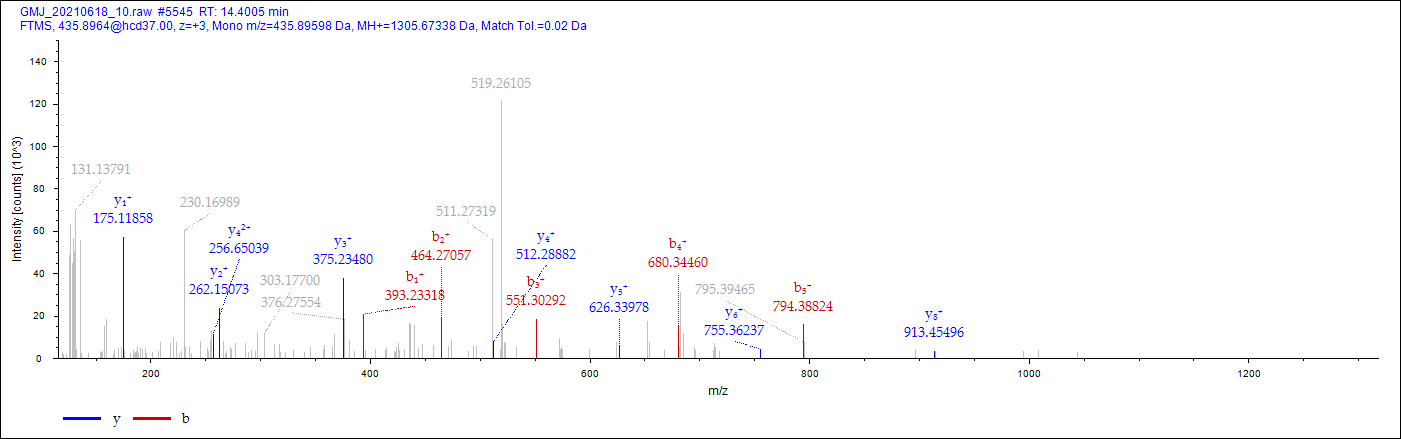
**

| **Dateset** | **Accession** | **Peptide** | **Spectar number** |
| --- | --- | --- | --- |
| **Table S5** | **P49753 [261-284]** | **[K].TMETLHLEYFEEAMNYLLSHPEVK.[G]** | **6** |

**
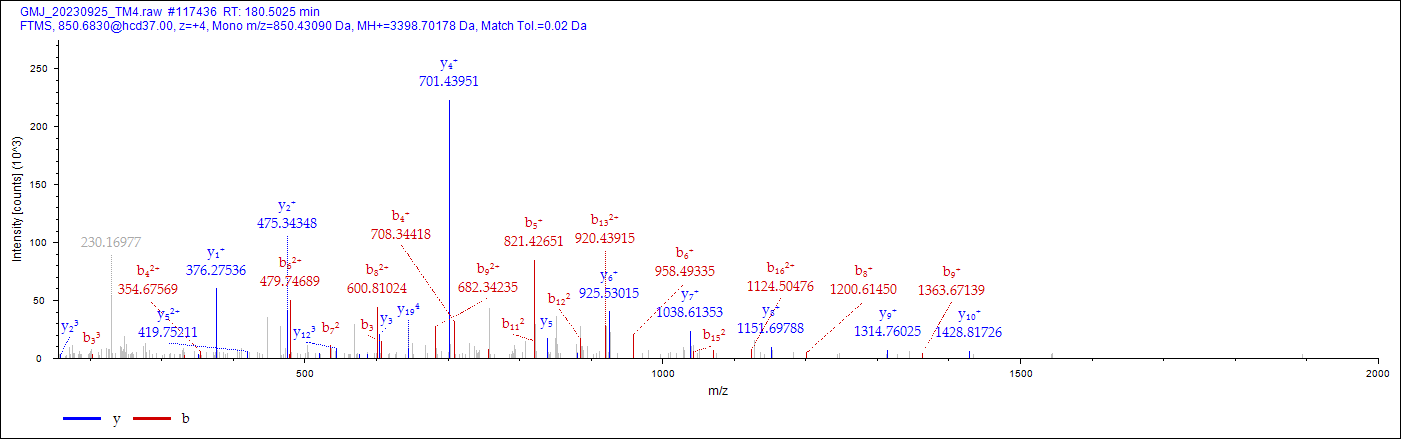

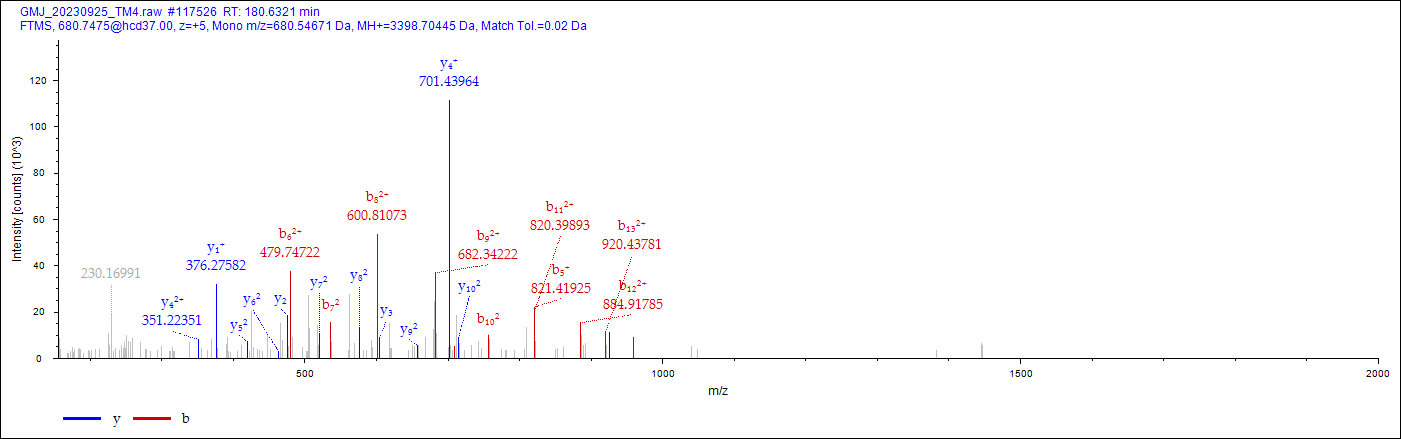

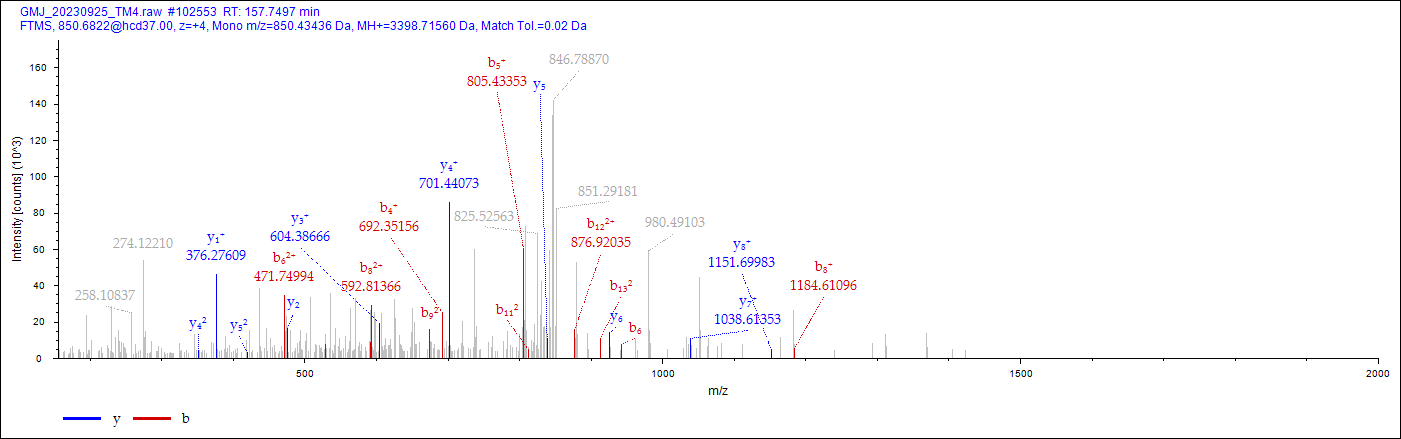

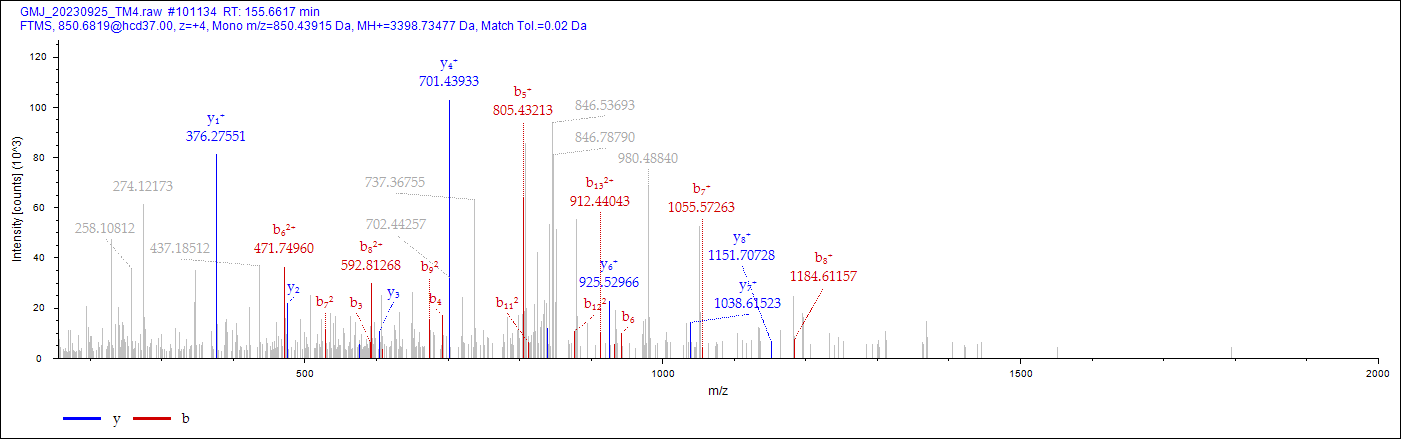

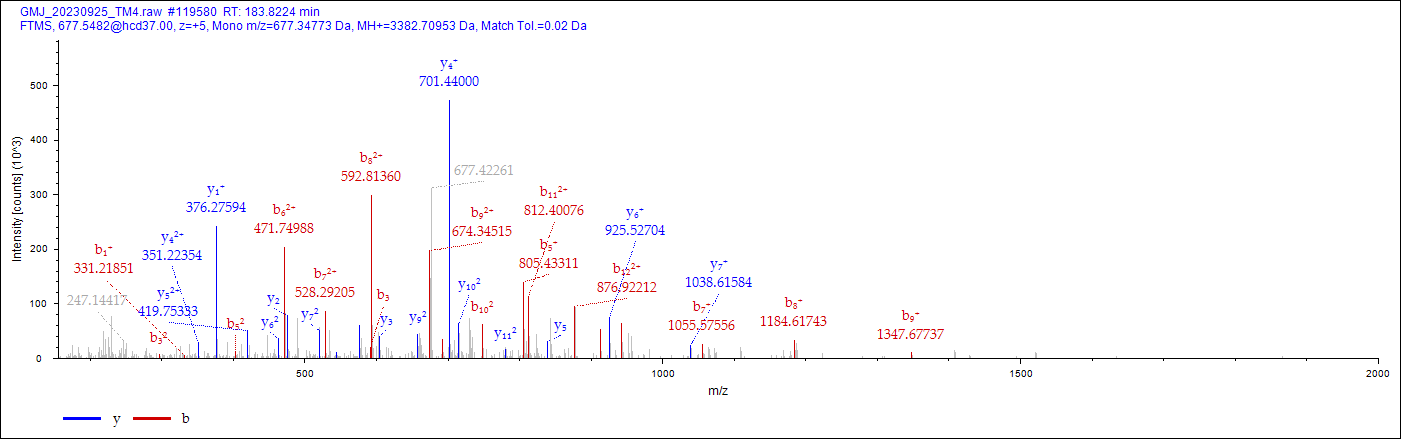

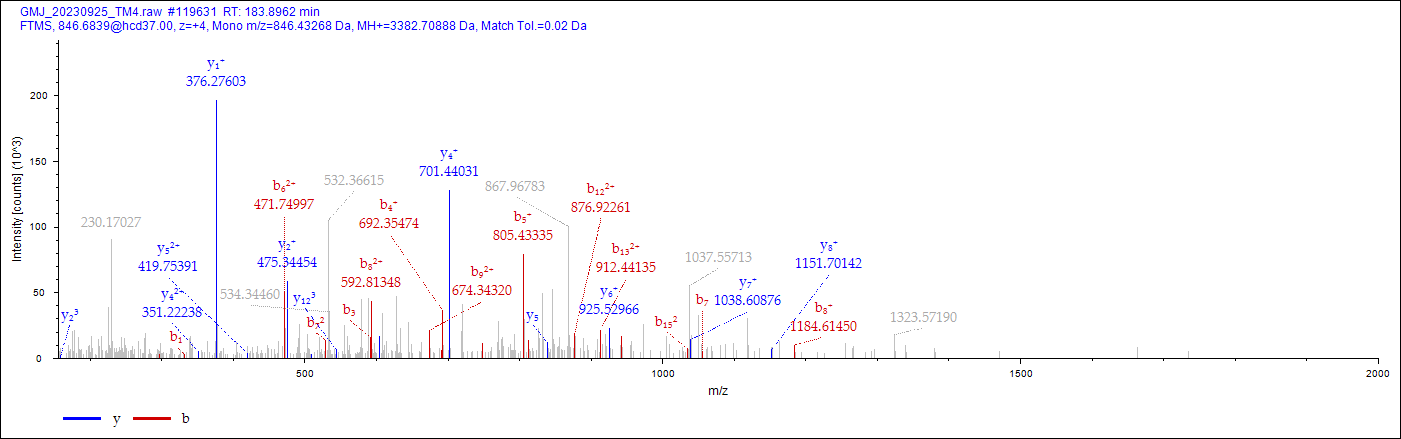
**

| **Dateset** | **Accession** | **Peptide** | **Spectar number** |
| --- | --- | --- | --- |
| **Table S5** | **P10176 [26-34]** | **[K].IHSLPPEGK.[L]** | **7** |

**
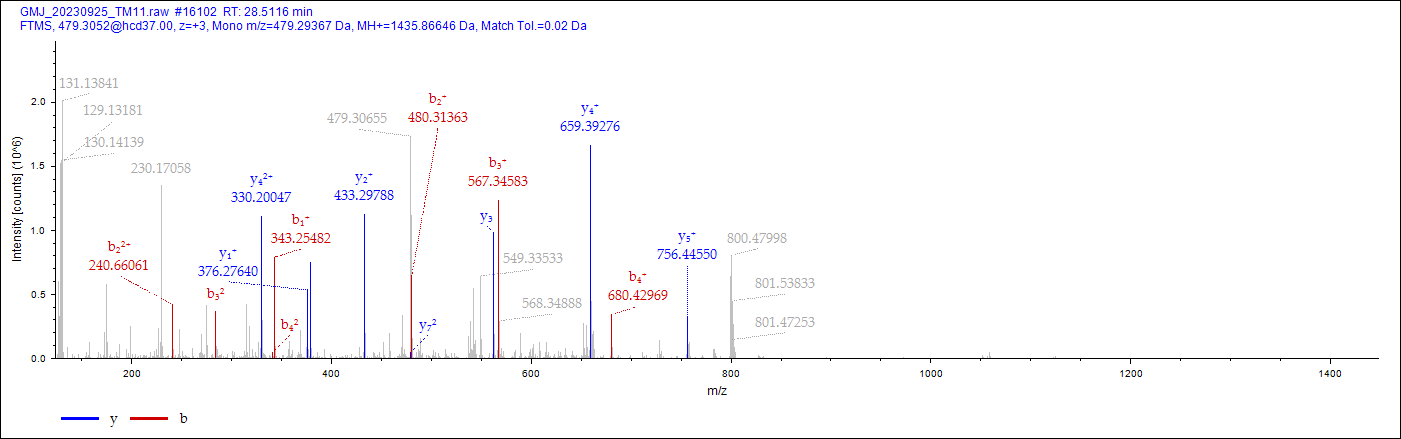

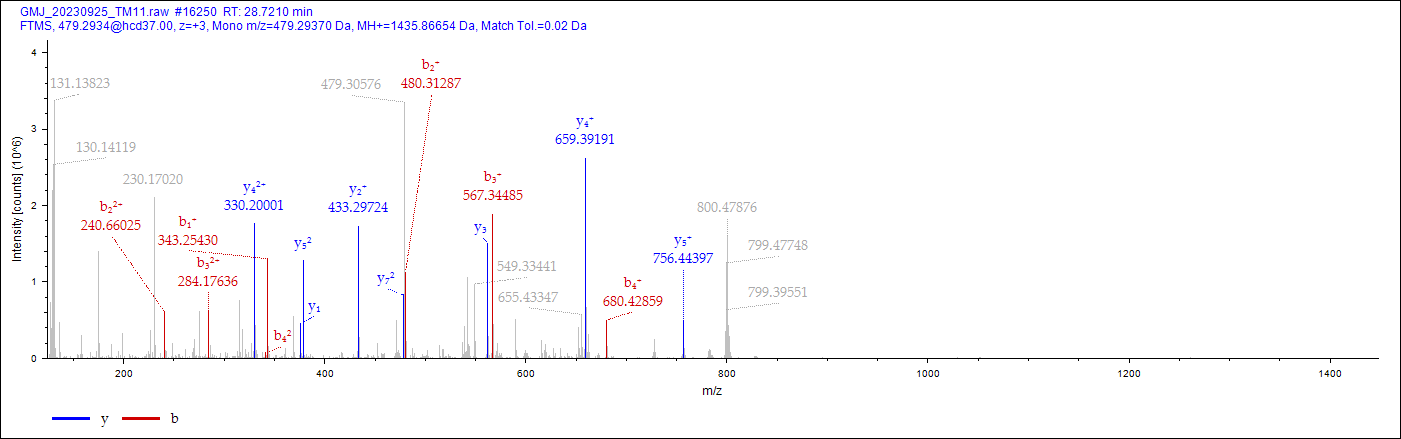

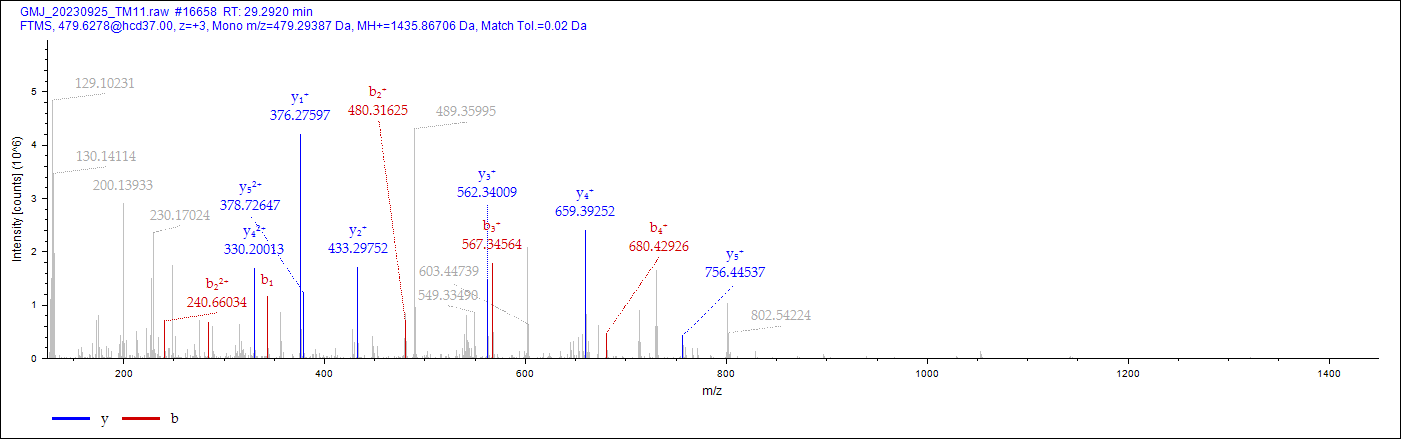

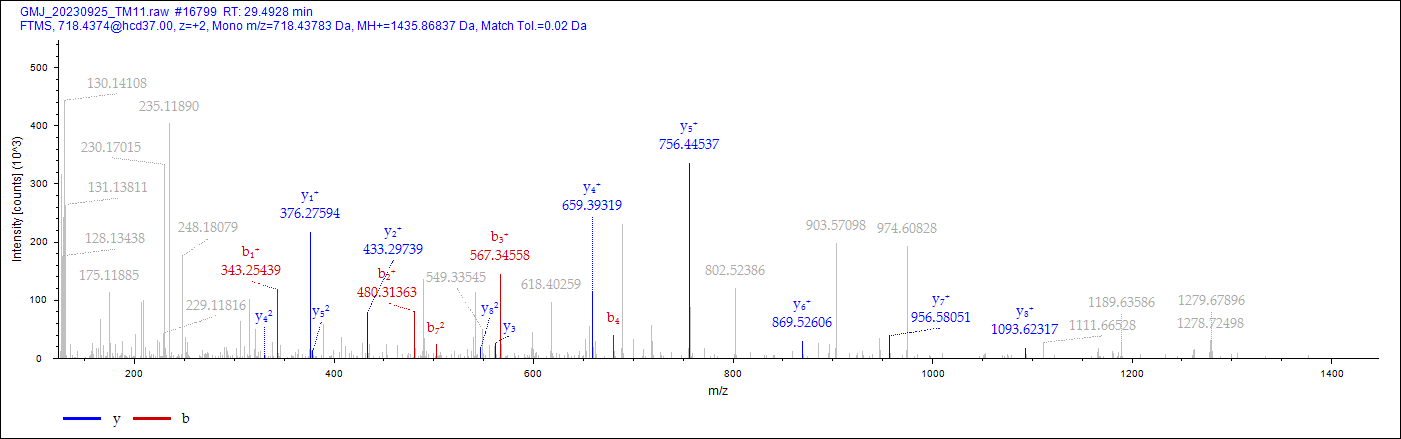

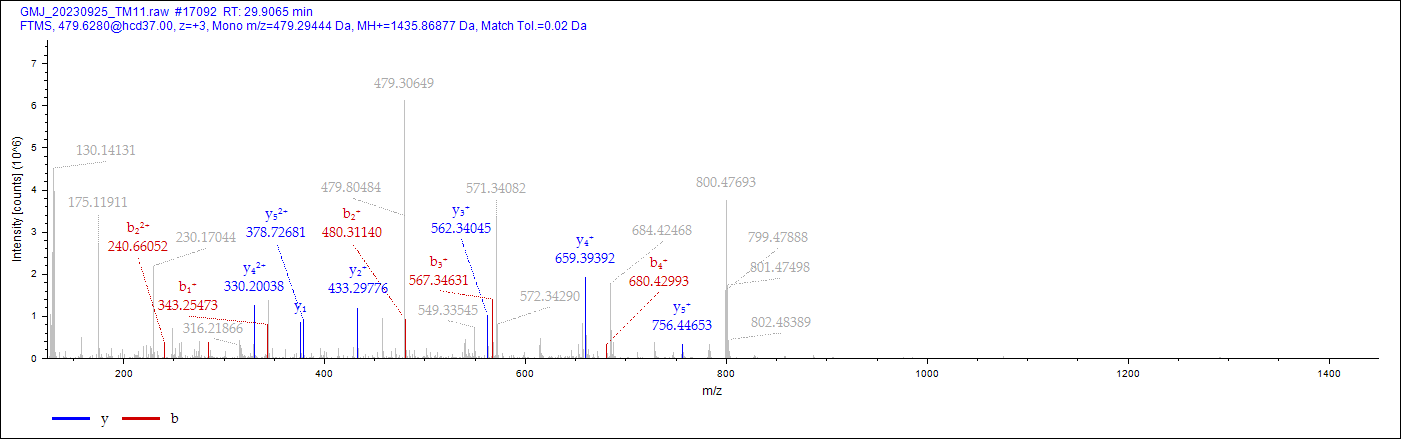

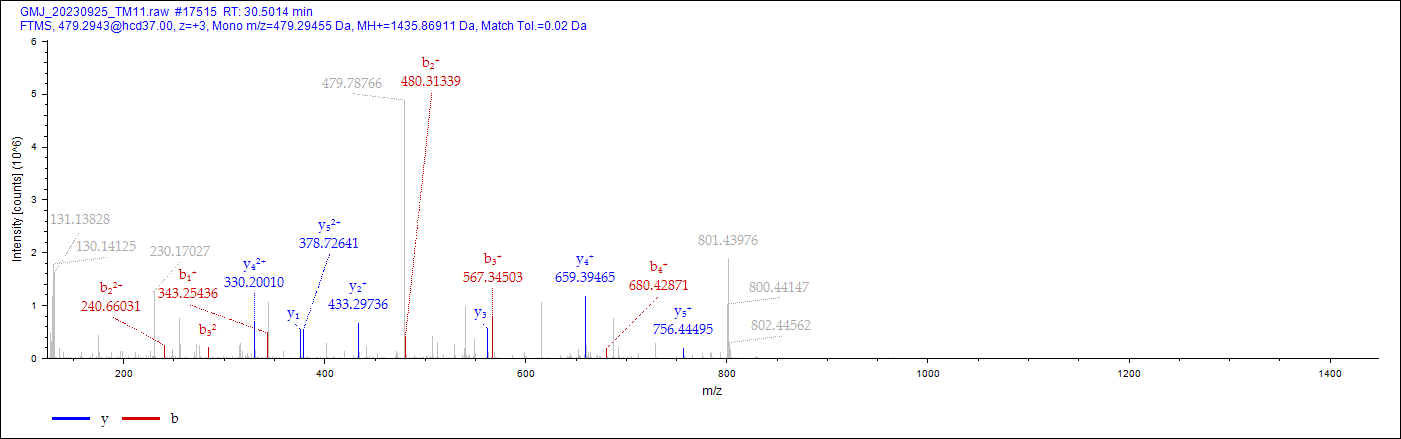

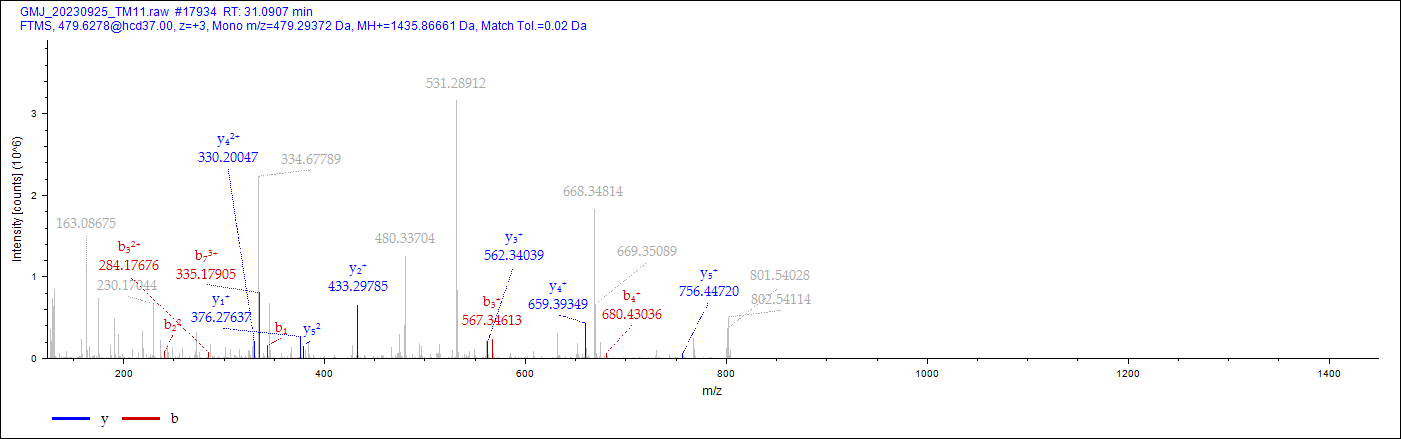
**

| **Dateset** | **Accession** | **Peptide** | **Spectar number** |
| --- | --- | --- | --- |
| **Table S5** | **Q96H96 [258-265]** | **[R].DDVLIGLK.[S]** | **1** |

**
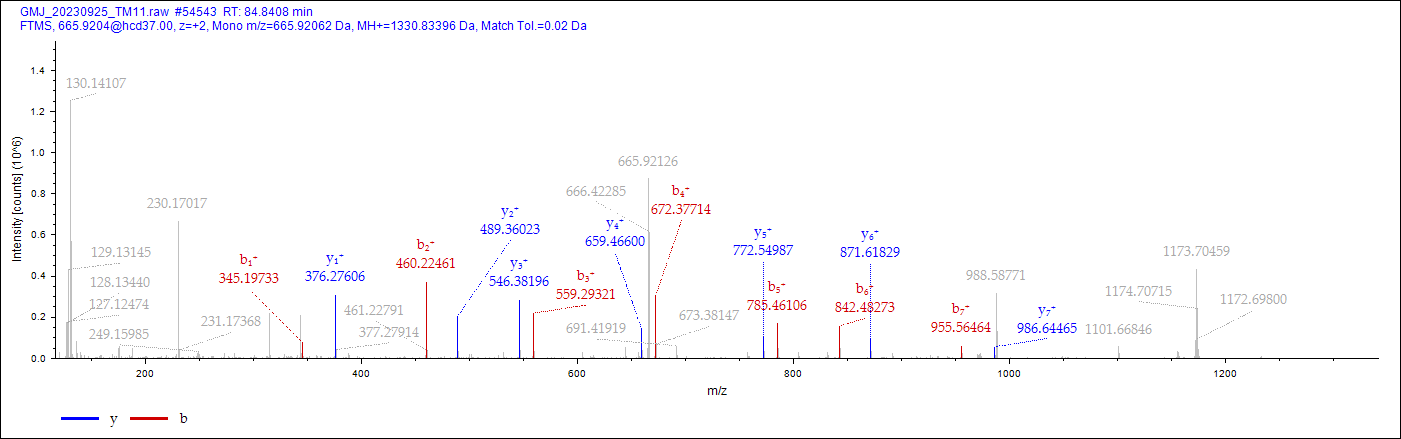
**
